# Effectiveness of Interactive Remote Rehabilitation After Total Knee Arthroplasty: Systematic Review and Meta-Analysis of Randomized Controlled Trials

**DOI:** 10.2196/89321

**Published:** 2026-08-07

**Authors:** Linbo Peng, Kexin Wang, Limin Wu, Yi Zeng, Bin Shen

**Affiliations:** 1 Department of Orthopedics, Orthopedic Research Institute, West China Hospital, Sichuan University, 37# Guoxue Road, Chengdu, Sichuan Province, 610041, China, 86 13881878767; 2 Sports Medicine Center, West China Hospital, Sichuan University, Chengdu, China; 3 Department of Clinical Research Management, Ministry of Science and Technology, West China Hospital, Sichuan University, Chengdu, China

**Keywords:** total knee arthroplasty, interactive remote rehabilitation, telerehabilitation, meta-analysis, randomized controlled trial

## Abstract

**Background:**

Remote rehabilitation has become increasingly relevant after total knee arthroplasty (TKA). However, existing reviews have often pooled interventions with markedly different levels of clinician involvement, making it difficult to determine whether interactive, feedback-enabled models provide distinct clinical value.

**Objective:**

This systematic review and meta-analysis evaluated the effectiveness of interactive remote rehabilitation (IRR), defined as technology-enabled rehabilitation involving bidirectional communication between patients and health care providers, compared with conventional rehabilitation after TKA.

**Methods:**

PubMed, Cochrane CENTRAL, Embase, Web of Science, CINAHL, Scopus, and CNKI were searched from inception to May 5, 2026. Randomized controlled trials enrolling adults after TKA and comparing IRR with conventional rehabilitation were eligible. Outcomes included pain, patient-reported function, range of motion, quadriceps muscle strength, mobility, general health status, and health-related quality of life. Risk of bias was assessed using the Cochrane Risk of Bias 2 tool. Random-effects meta-analyses used Hartung-Knapp-Sidik-Jonkman CIs and Nagashima-corrected 95% prediction intervals (PIs); analyses were stratified as short-, mid-, and long-term follow-up. The protocol was prospectively registered in PROSPERO (CRD420251049015).

**Results:**

In total, 23 randomized controlled trials involving 2607 participants were included. A total of 7 studies were judged to be at low risk of bias, 7 raised some concerns, and 9 were at high risk of bias. Across most primary and secondary outcomes, IRR did not show statistically significant advantages over conventional rehabilitation, including pain, Western Ontario and McMaster Universities Osteoarthritis Index, Knee Injury and Osteoarthritis Outcome Score, Timed Up and Go test, and EQ-5D outcomes, across follow-up periods. A very small benefit was observed for short-term active extension range of motion (mean difference 0.26, 95% CI 0.04-0.48; 95% PI 0.00-0.52), which remained statistically significant after excluding high-risk studies but was of limited clinical magnitude. Short-term quadriceps muscle strength favored IRR in the primary analysis (standardized mean difference 0.60, 95% CI 0.01-1.19; 95% PI −0.63 to 1.87), but the effect was not robust after sensitivity analysis, and the PI crossed the null. Evidence for 36-Item Short Form Survey and 6-minute walk test outcomes was insufficient for quantitative synthesis. Heterogeneity was substantial for several outcomes, PIs were frequently wide, and certainty of evidence was generally low to very low.

**Conclusions:**

This review is innovative in focusing specifically on bidirectional, IRR rather than treating all remote or technology-assisted rehabilitation as a single category, and it differs from prior reviews by combining this conceptual distinction with time-stratified analyses and more conservative random-effects inference. This synthesis brings to the field a clearer and more clinically interpretable assessment of what IRR currently adds after TKA: available evidence does not establish superiority over conventional rehabilitation, although IRR may represent a feasible care model where access to in-person rehabilitation is limited. These findings can inform service planning and trial design while underscoring the need for higher-quality studies to identify which interactive features, patient groups, and contexts are most likely to benefit.

## Introduction

Total knee arthroplasty (TKA) is a well-established surgical intervention for end-stage knee osteoarthritis and is widely recognized for its ability to relieve pain and improve joint function [1]. However, optimal postoperative recovery does not depend on surgery alone [2]. Rehabilitation after TKA is essential for reducing pain, restoring range of motion (ROM), rebuilding lower-limb strength, improving mobility, and facilitating return to daily activities and social participation [3,4]. This recovery process usually extends beyond the inpatient period and requires sustained exercise participation, appropriate progression of training intensity, and timely correction of movement patterns [5]. In routine care, however, access to structured rehabilitation may be constrained by travel distance, uneven distribution of rehabilitation resources, financial and time burdens, and difficulty maintaining adherence once patients return home [6,7]. These barriers have stimulated interest in alternative models that can extend rehabilitation support beyond conventional face-to-face settings [8].

The rapid development of telecommunication technologies has created new opportunities for delivering postoperative rehabilitation remotely [9]. Remote rehabilitation may be implemented through videoconferencing, mobile apps, wearable sensors, web-based platforms, or combinations of these approaches [10]. In principle, such systems can reduce geographical barriers, support home-based recovery, and facilitate more flexible care delivery while maintaining some degree of professional oversight [11]. Importantly, remote rehabilitation is not a single uniform intervention. Some programs merely provide exercise instructions or educational materials for independent completion, whereas others incorporate real-time supervision, asynchronous feedback, data monitoring, goal adjustment, or repeated patient-clinician communication [12]. These differences are clinically meaningful because the therapeutic value of a remote program may depend not only on where care is delivered, but also on whether patients receive timely guidance, corrective feedback, and individualized reinforcement during recovery [13].

Several systematic reviews and meta-analyses have evaluated the effectiveness of remote rehabilitation or telerehabilitation following TKA [14-18]. Overall, these studies have generally suggested that remote rehabilitation may achieve outcomes comparable to conventional in-person rehabilitation for pain and functional recovery, with potential advantages related to convenience, accessibility, and cost. Other reviews have focused on more specific digital modalities, such as virtual reality–based rehabilitation or smart device–assisted telerehabilitation, and have reported possible benefits in selected outcomes [6,17]. Nevertheless, the existing literature remains difficult to interpret from an intervention-design perspective. Most prior reviews have classified interventions according to their mode of delivery, such as telerehabilitation, remote rehabilitation, or home-based digital rehabilitation, or according to the technological platform used, rather than using therapeutic interactivity as the primary conceptual criterion for intervention definition.

As a result, interventions with substantially different levels of clinician involvement are frequently synthesized within the same evidence base. For example, highly interactive programs that provide real-time visual supervision, sensor-based monitoring, or individualized adjustment may be pooled together with minimally supervised programs that rely primarily on prerecorded exercise videos or app-based self-management [19]. Although both may be described as remote rehabilitation, they differ considerably in the extent to which they reproduce the therapeutic functions of face-to-face rehabilitation. Similarly, technology-centered reviews may clarify whether a particular tool, such as virtual reality or wearable devices, appears useful, but they do not directly address whether bidirectional communication itself contributes to treatment effectiveness [20,21]. This distinction matters because interactivity may represent a core rehabilitative mechanism rather than a superficial technical characteristic. Bidirectional communication enables clinicians to monitor progress, identify poor movement execution, reinforce adherence, adjust rehabilitation plans, and respond to patient concerns, all of which may influence postoperative outcomes [22,23].

Despite the potential importance of this distinction, the effectiveness of interactive remote rehabilitation (IRR) after TKA has not been comprehensively evaluated. In this review, IRR refers to technology-enabled rehabilitation programs that involve bidirectional communication between patients and health care providers, whether delivered synchronously, asynchronously, or through hybrid models [24,25]. This conceptual framing differs from previous reviews that have treated remote rehabilitation as a relatively homogeneous category. It also reflects current developments in digital care, as contemporary rehabilitation systems increasingly combine apps, teleconsultation, wearable monitoring, automated data transmission, and clinician feedback rather than relying on a single delivery format [26,27]. In recent years, multiple randomized controlled trials (RCTs) have examined such interactive approaches after TKA, including app-based rehabilitation systems, sensor-assisted monitoring platforms, cloud-based follow-up systems, and hybrid feedback models [28-32]. These trials have also reported a broader range of postoperative outcomes, including pain, patient-reported function, ROM, muscle strength, mobility, health-related quality of life, satisfaction, and health care use, making a more comprehensive reassessment of the evidence timely and necessary.

A further limitation of prior syntheses is that postoperative effects are often summarized without sufficient distinction between follow-up periods [28-32]. However, the goals and content of rehabilitation evolve across recovery, with earlier phases generally focusing on symptom control and restoration of joint motion, and later phases increasingly emphasizing strength, mobility, and broader functional recovery [33]. Time-stratified synthesis may therefore provide a more clinically meaningful assessment of IRR than pooling outcomes measured at distinct stages of postoperative rehabilitation.

Given these conceptual and methodological gaps, a focused synthesis of IRR after TKA is warranted. This systematic review and meta-analysis therefore aimed to evaluate the effectiveness of IRR compared with conventional rehabilitation after TKA, restricting inclusion to RCTs and examining outcomes across prespecified short-, mid-, and long-term follow-up periods. Unlike earlier reviews, this study treats interactivity—rather than remote delivery alone—as the defining intervention feature. By combining this more precise conceptual framework with comprehensive database searching, updated randomized evidence, time-stratified outcome analysis, and conservative meta-analytic methods, this review seeks to provide a clearer and more clinically interpretable assessment of what IRR currently contributes to postoperative recovery after TKA.

## Methods

### Protocol and Registration

This systematic review and meta-analysis was conducted and reported in accordance with the PRISMA (Preferred Reporting Items for Systematic Reviews and Meta-Analyses) 2020 statement [34]. The protocol was prospectively registered in PROSPERO (CRD420251049015). The PRISMA 2020 checklist is provided in Checklist 1.

### Eligibility Criteria

We included RCTs enrolling adult patients (≥18 years) undergoing TKA that compared IRR with any control intervention, including face-to-face rehabilitation, unsupervised home-based rehabilitation, usual care, outpatient rehabilitation, or inpatient rehabilitation.

IRR was defined as rehabilitation programs delivered through digital or telecommunication technologies that enabled bidirectional communication between patients and health care providers. These interventions could include synchronous modalities (eg, real-time video or telephone-based supervision), asynchronous modalities (eg, app-based programs with delayed feedback), or hybrid models combining both approaches.

We excluded nonrandomized studies, non-IRR interventions (ie, programs without clinician-patient interaction), studies involving mixed joint replacement populations without separate TKA data, and studies with insufficient data for quantitative synthesis.

Only studies published in English or Chinese were included.

### Information Sources

We systematically searched PubMed (MEDLINE), Cochrane CENTRAL, Embase (via Ovid), Web of Science Core Collection, CINAHL, Scopus, and CNKI from database inception to May 5, 2026 (updated from the original search conducted up to May 25, 2025). In addition, the reference lists of included studies and relevant systematic reviews were manually screened to identify potentially eligible studies.

### Search Strategy

The search strategy was developed and reported in accordance with PRISMA-S (Preferred Reporting Items for Systematic Reviews and Meta-Analyses Literature Search Extension), incorporating both controlled vocabulary and free-text terms related to TKA and remote rehabilitation [35]. The search strategy was developed by 2 authors (LP and BS) and independently checked by 2 additional authors (KW and YZ) for completeness, consistency, and relevance to the review question. For database-specific searches, we used MeSH terms in PubMed, Emtree terms in Embase, database-specific subject headings where applicable, field modifiers, truncation, and a broad range of synonyms related to TKA, telerehabilitation, remote rehabilitation, digital rehabilitation, telehealth, app-based rehabilitation, virtual rehabilitation, and rehabilitation therapy. The PRISMA-S checklist is provided in Checklist 2, and the PRISMA-S Search Reporting Details and Complete Database-Specific Search Strategies checklist is provided in Checklist 3.

### Study Selection

Two reviewers (LP and KW) independently screened records in 2 stages (title and abstract screening followed by full-text review) using EndNote (version 21; Clarivate) and Rayyan (Rayyan Systems Inc). Duplicates were removed prior to screening. Studies were assessed against predefined eligibility criteria. Disagreements were resolved through discussion or by consulting a senior reviewer.

### Data Extraction

Two reviewers (LW and YZ) independently extracted data using a standardized, pilot-tested data collection form, including study characteristics (first author, year, country, and study design), sample size, intervention and control descriptions, type of remote intervention (synchronous, asynchronous, or hybrid), intervention details, treatment duration, follow-up time points, and outcome measures (eg, pain, functional outcomes, ROM, muscle strength, mobility, general health status, and health-related quality of life).

Where necessary, data were extracted or calculated from reported statistics. For trials with multiple publications, we used the earliest report as the primary source and supplemented it with additional follow-up data from subsequent publications.

### Outcome Definitions

Outcomes were prespecified and categorized into domains prior to analysis. The primary outcomes included pain (eg, visual analog scale [VAS] and numeric rating scale [NRS]) and functional outcomes (eg, Western Ontario and McMaster Universities Osteoarthritis Index [WOMAC] and Knee Injury and Osteoarthritis Outcome Score [KOOS]), while secondary outcomes included ROM, quadriceps muscle strength, mobility (eg, Timed Up and Go test [TUG] and 6-minute walk test [6MWT]), general health status (36-Item Short Form Survey [SF-36]), and health-related quality of life (EQ-5D). Outcomes were grouped and synthesized based on their underlying constructs and measurement characteristics. When multiple studies assessed the same construct using comparable or transformable scales (eg, pain measured by VAS or NRS), results were pooled. In contrast, outcomes representing distinct domains (eg, patient-reported function, mobility, general health status, and health-related quality of life) or measured using instruments with substantially different conceptual frameworks (eg, WOMAC, KOOS, TUG, and 6MWT) were analyzed separately to preserve clinical interpretability and avoid inappropriate aggregation.

Outcomes were analyzed at predefined follow-up intervals: short-term (≤6 weeks), mid-term (>6 weeks to ≤3 months), and long-term (>3 months). When multiple time points were reported within the same interval, the time point closest to the upper limit of the predefined interval was selected. For long-term outcomes, given the limited availability of data beyond 12 months, the time point closest to 6 months was preferentially selected to ensure comparability across studies and to capture clinically stable treatment effects. A single time point per category was selected to avoid double counting.

### Risk of Bias Assessment

Risk of bias was assessed independently by 2 reviewers (LP and BS) using the Cochrane Risk of Bias 2 tool across 5 domains: bias arising from the randomization process, bias due to deviations from intended interventions, bias due to missing outcome data, bias in measurement of the outcome, and bias in selection of the reported result [36].

### Data Synthesis and Statistical Analysis

We performed meta-analyses using R (version 4.0.0; R Foundation for Statistical Computing). For continuous outcomes, mean difference (MD) or standardized mean difference (SMD) with 95% CIs were calculated, depending on the measurement scales used. For dichotomous outcomes, risk ratios with 95% CIs were calculated. In addition, outcome measures were synthesized based on their underlying constructs. When multiple instruments assessed a similar construct and were considered conceptually comparable, results were pooled using SMD. In contrast, outcomes representing distinct domains or measured using instruments with different conceptual frameworks (eg, patient-reported function vs performance-based mobility) were analyzed separately to preserve clinical interpretability and avoid inappropriate aggregation.

Between-study heterogeneity was assessed using the *I*^2^ statistic and interpreted in conjunction with clinical and methodological considerations. A random-effects model was applied to all meta-analyses to account for expected between-study variability [37]. For random-effects analyses, 95% prediction intervals (PIs) were additionally calculated to estimate the range of true effects across different settings [38]. CIs were interpreted as reflecting uncertainty around the pooled average effect, whereas PIs were interpreted as reflecting the expected distribution of true effects across comparable clinical settings [38,39]. PIs were calculated using the confidence distribution approach proposed by Nagashima et al [39] to improve interval estimation in random-effects meta-analysis, particularly when the number of included studies was small.

For outcomes with a small number of studies, the Hartung-Knapp-Sidik-Jonkman (HKSJ) method was used to provide more robust estimates [40]. Sensitivity analyses were conducted by excluding studies judged to be at high risk of bias. Small-study effects and potential publication bias were assessed using funnel plots and the Egger test when at least 10 studies were available [41]. When the number of included studies was insufficient (eg, fewer than 10 studies for publication bias assessment or limited data for subgroup analysis), these analyses were not performed [42]. A narrative synthesis was conducted when meta-analysis was not feasible. To avoid unit-of-analysis errors, we ensured that each study contributed independent participant data and that no double counting occurred across comparisons [43].

### Certainty of Evidence

The certainty of evidence for each outcome was assessed using the GRADE (Grading of Recommendations Assessment, Development and Evaluation) approach [44]. The overall quality of evidence was evaluated across studies, considering risk of bias, inconsistency, indirectness, imprecision, and publication bias.

Each outcome was rated as high, moderate, low, or very low certainty. The assessment was conducted independently by 2 reviewers, with disagreements resolved through discussion or consultation with a senior reviewer.

## Results

### Study Selection and Characteristics

A total of 5154 records were identified through database searches, including PubMed (n=876), CENTRAL (n=1010), Embase via Ovid (n=1730), Web of Science (n=395), CINAHL (n=126), Scopus (n=931), and CNKI (n=86). An additional 12 records were identified through manual screening of reference lists.

After removal of 2289 duplicates, 2865 records remained for title and abstract screening. Of these, 2639 records were excluded based on predefined eligibility criteria. The remaining 226 full-text papers were assessed for eligibility.

Among the full-text papers, 203 were excluded for the following reasons: not RCTs (n=68), absence of IRR interventions (n=73), not involving TKA or involving mixed joint populations without separate TKA data (n=27), and insufficient data for extraction (n=35).

**Figure 1.**
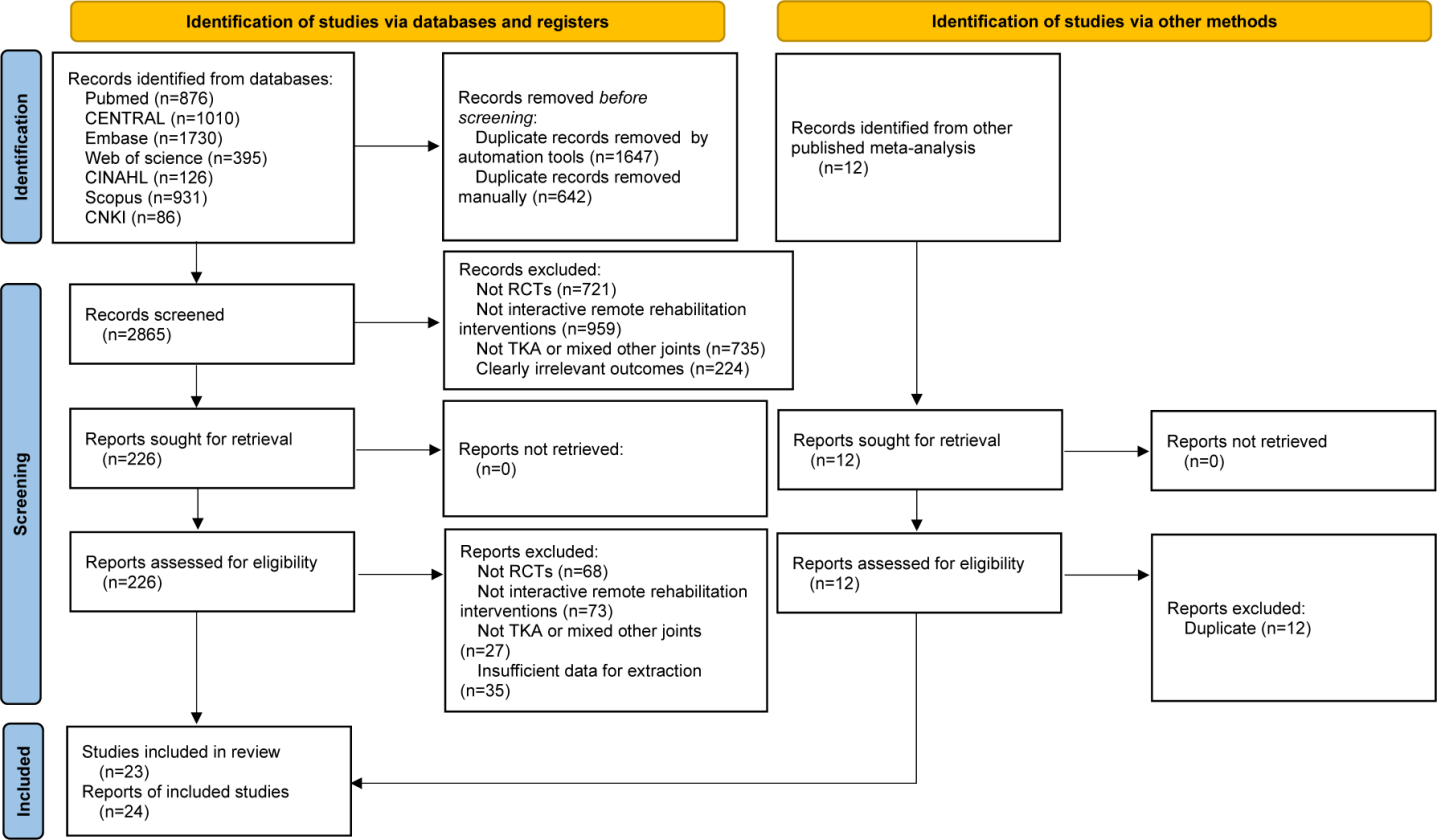
PRISMA (Preferred Reporting Items for Systematic Reviews and Meta-Analyses) flow diagram of study selection process. RCT: randomized controlled trial; TKA: total knee arthroplasty.

Ultimately, 24 reports describing 23 RCTs [28-32,45-62] were included in the qualitative synthesis and quantitative meta-analysis. The PRISMA flow diagram of the study selection process is presented in Figure 1.

The included RCTs were published between 2003 and 2025, with sample sizes ranging from 20 to 368 participants. Studies were conducted across Asia (China, Korea, Singapore, and Pakistan), Europe (Spain and the Netherlands), North America (the United States and Canada), and Australia, reflecting a broad international distribution. Intervention durations varied from 2 weeks to 6 months, with follow-up assessments extending to 12 months in some trials.

All included interventions were consistent with the predefined definition of IRR, involving bidirectional communication between patients and health care providers. Intervention modalities included synchronous real-time telerehabilitation (eg, videoconferencing), asynchronous platforms (eg, app-based exercise programs with delayed feedback), and hybrid approaches. Some interventions incorporated wearable sensors, motion analysis systems, or app-based monitoring.

Control groups were heterogeneous and included conventional in-person rehabilitation, unsupervised home exercise programs, outpatient rehabilitation, inpatient rehabilitation, and usual care.

Outcomes were categorized into predefined domains. Pain outcomes were commonly assessed using the VAS and NRS. Functional outcomes were evaluated using various scales, including WOMAC, KOOS, and Hospital for Special Surgery Knee Score, as well as performance-based measures such as the TUG and 6MWT. Additional outcomes included ROM, quadriceps muscle strength, general health status (SF-36), and health-related quality of life (EQ-5D), patient satisfaction, and health care use. Detailed characteristics of the included studies are summarized in Table 1.

**Table 1.** Summary of included randomized controlled trials on remote rehabilitation after total knee arthroplasty.

Author (year)	Country	Sample size (intervention vs control group), n	Type of remote intervention	Intervention group	Control group	Treatment duration	Outcome assessment time points	Main outcomes
Liu (2011) [45]	China	Intervention: 42 Control: 42	Synchronous	Synchronous video-based rehabilitation via SharevisionPC-3000 with real-time audio-visual interaction	Usual care	6 months	3 and 6 months	HSS^a^ and SF-36^b^
Russell et al (2011) [46]	Australia	Intervention: 31 Control: 34	Synchronous	Computer-based telerehabilitation system via videoconferencing	Usual care	6 weeks	6 weeks	VAS^c^ WOMAC^d^ (pain, stiffness, function, and total) ROM^e^ (active flexion, passive flexion, and extension) Quadriceps muscle strength Limb girth (knee and calf) TUG^f^ Patient-Specific Functional Scale Gait Assessment Rating Scale
Piqueras et al (2013) [47]	Spain	Intervention: 68 Control: 65	Synchronous	Interactive virtual telerehabilitation sessions (wireless sensors and wireless sensors and web portal for the therapist)	Face-to-face rehabilitation	2 weeks	2 weeks and 3 months	VAS ROM (active flexion and active extension) Quadriceps muscle strength Hamstring muscle strength TUG
Moffet et al (2015) [48]	Canada	Intervention: 84 Control: 98	Synchronous	Telerehabilitation via videoconference	Face-to-face rehabilitation	2 months	2 and 4 months	WOMAC (pain, stiffness, function, and total) KOOS^g^ (symptoms, pain, activities of daily living, sports and recreational activities, and quality of life) 6MWT^h^ TST^i^ ROM (flexion and extension) knee strength
Bini and Mahajan (2017) [50]	United States	Intervention: 14 Control: 15	Asynchronous	Telerehabilitation via instructional videos on iPod touch with feedback	Outpatient standard rehabilitation	3 months	3 months	VAS KOOS (total) VR-12^j^
Sun and Sun (2017) [49]	China	Intervention: 60 Control: 60	Asynchronous	Telerehabilitation via instructional videos	Usual care	12 weeks	1 week, 3, 6, 9, and 12 weeks	WOMAC (pain, stiffness, and function) ROM (active ROM and total)
Zhao et al (2018) [51]	China	Intervention: 60 Control: 60	Synchronous	Telerehabilitation via app-guided exercise	Usual care	3 months	1 month and 3 months	WOMAC (total) HSS (total) ROM (active and passive ROM, total)
Prvu Bettger et al (2020) [54]	United States	Intervention: 143 Control: 144	Synchronous	Telerehabilitation with virtual exercise rehabilitation assistant	Usual care	12 weeks	6 and 12 weeks	Total health-care costs (12 weeks) KOOS (symptoms, pain, activities of daily living, sports and recreational activities, and quality of life, total; 6 and 12 weeks) KOOS JR^k^ (6 and 12 weeks) ROM (flexion and extension; 6 weeks) VAS (6 weeks) 10-m gait speed (6 weeks) Falls, pain, and hospital readmissions (12 weeks) PROMIS^l^
Timmers et al (2019) [52]	Netherlands	Intervention: 114 Control: 99	Synchronous	Mobile app with pre- or postoperative content and telerehabilitation	Mobile app with basic information	4 weeks	1 week, 2, 3, and 4 weeks	NRS^m^ (1, 2, 3, and 4 weeks) KOOS PS^n^ (total, 4 weeks) EQ-5D (4 weeks)
Bell et al (2020) [53]	United States	Intervention: 10 Control: 10	Synchronous	Outpatient rehabilitation and home exercise based on telerehabilitation	Outpatient rehabilitation and home exercise	10 weeks	5 and 10 weeks	NRS (pain) Activities of daily living (total) VR-12 (total) ROM (active flexion and active extension) 6MWT Stair climbing test Unilateral balance test TUG
Crawford^o^ et al (2021) [56]	United States	Intervention: 160 Control: 185	Synchronous	App-based remote rehabilitation	Standard of care with formal physiotherapy	12 weeks	1 month, 3 months, 6 months, and 1 year	ROM (passive flexion; 1 month and 3 months) KOOS (1 month, 3 months, 6 months, and 1 year) EQ-5D-5L (1 month, 3 months, 6 months, and 1 year) Single leg stance (1 month and 3 months) TUG
Gu (2021) [55]	China	Intervention: 34 Control: 34	Synchronous	App-based remote rehabilitation	Usual care	6 months	3 and 6 months	Quadriceps muscle strength HSS (pain, function, ROM, muscle strength, flexion deformity, and instability) MOS SF-36^p^
Duong et al (2023) [57]	Australia	Intervention: 51 Control: 51	Synchronous	App-based remote rehabilitation	Usual care	3 months	3, 6, and 12 months	NRS (3, 6, and 12 months) Pain Disability Index (3, 6, and 12 months) Participation in physical activity (3, 6, and 12 months) Sedentary behavior AQoL-8D^q^
Shim et al (2023) [58]	Korea	Intervention: 27 Control: 27	Synchronous	Telemonitoring+online education	conventional rehabilitation	12 weeks	3, 12, and 24 weeks	NRS (pain) ROM (active ROM, total) WOMAC (total) EQ-5D-5L BBS^r^ 4-m gait speed Quadriceps muscle strength Hamstring muscle strength
Bradbury et al (2024) [28]	United States	Intervention: 95 Control: 102	Synchronous	App-based remote rehabilitation	Outpatient physical therapy	6 weeks	6, 12, and 52 weeks	NRS (6 and 12 weeks) ROM (extension and flexion; 6 weeks) KOOS JR, VR-12 MCS^s^, VR-12 PCS^t^, TUG (6 weeks) 4-m gait speed (6 weeks)
Nuevo et al (2024) [30]	Spain	Intervention: 23 Control: 22	Synchronous	App-based remote rehabilitation and real-time feedback (a web platform and an inertial motion sensor)	Conventional rehabilitation	4 weeks	2 and 4 weeks	VAS (pain) ROM (active and passive flexion and extension) WOMAC (total) TUG Quadriceps muscle strength Hamstring muscle strength EQ-5D-5L questionnaire, EQ-5D-5L VAS
Pua et al (2024) [31]	Singapore	Intervention: 47 Control: 44	Asynchronous	Unsupervised app-based remote rehabilitation tablet and sensor and monitor	Outpatient rehabilitation	10 weeks	12 and 24 weeks	Pain ROM (flexion and extension) Fast gait speed KOOS (total) 30-second sit-to-stand test Quadriceps muscle strength sit-to-stand test
Han and Kong (2024) [29]	Korea	Intervention: 14 Control: 14	Synchronous	App-based remote rehabilitation	Conventional rehabilitation	12 weeks	2 and 3 months	VAS ROM (passive, total) WOMAC (total) isokinetic knee muscle strength
Zhao et al (2024) [32]	China	Intervention: 50 Control: 50	Synchronous	App-based remote rehabilitation and wearable sensors	Home-based rehabilitation	12 weeks	2, 6, and 12 weeks	VAS WOMAC (total) KSS^u^ (total) ROM (flexion) SF-36 SLST^v^ 5XSST^w^
Chen et al (2025) [59]	China	Intervention: 76 Control: 75	Asynchronous	Cloud-based follow-up system	Conventional rehabilitation	12 weeks	2, 4, and 12 weeks	WOMAC (total) SF-36 (total)
Cui et al (2025) [62]	China	Intervention: 40 Control: 40	Asynchronous	App-based remote rehabilitation	Conventional rehabilitation	6 months	1 month, 3 and 6 months	WOMAC (total), Quality of life (WOMAC) ROM (active flexion and active extension)
Jung et al (2025) [60]	Korea	Intervention: 24 Control: 25	Hybrid	App-based remote rehabilitation and wearable sensors	Conventional rehabilitation	12 weeks	3, 6, and 12 weeks	ROM (total) WOMAC (pain, stiffness, and function) EQ-5D-5L KOOS (pain, stiffness, sport function, activities of daily living, and quality of life) Gait speed and gait ROM
Sadiq et al (2025) [61]	Pakistan	Intervention: 22 Control: 22	Asynchronous	Web-based telerehabilitation	Conventional rehabilitation	22 weeks	14 and 22 weeks	KOOS (pain, symptoms, sport function, activities of daily living, and quality of life) EQ-5D-5L BBS Joint position sense

a HSS: the Hospital for Special Surgery Knee Score.

b SF-36: 36-Item Short Form Survey.

c VAS: visual analog scale.

d WOMAC: Western Ontario and McMaster Universities Osteoarthritis Index.

e ROM: range of motion.

f TUG: Timed Up and Go test.

g KOOS: Knee Injury and Osteoarthritis Outcome Score.

h 6MWT: 6-minute walk test.

i TST: Timed stair test.

j VR-12: Veterans RAND 12-Item Health Survey.

k KOOS JR: Knee Injury and Osteoarthritis Outcome Score for Joint Replacement.

l PROMIS: Patient-Reported Outcomes Measurement Information System.

m NRS: numeric rating scale.

n KOOS PS: Knee Injury and Osteoarthritis Outcome Score Physical Function.

o This study represents a follow-up report of the same randomized controlled trial, providing long-term outcome data [63].

p MOS SF-36: Medical Outcomes Study 36-Item Short Form Health Survey.

q AQoL-8D: Assessment of Quality of Life-8 Dimensions.

r BBS: Berg Balance Scale.

s VR-12 MCS: Veterans RAND 12-Item Health Survey Mental Component Score.

t VR-12 PCS: Veterans RAND 12-Item Health Survey Physical Component Summary.

u KSS: Knee Society Score.

v SLST: Single-Leg Stance Test.

w 5XSST: Five-Times Sit-to-Stand Test.

### Risk-of-Bias Assessment

Using the Cochrane Risk of Bias 2 tool, a total of 23 RCTs were included in the risk of bias assessment. Overall, 7 studies were judged as low risk of bias, 7 studies raised some concerns, and the remaining 9 studies were considered to have a high risk of bias.

**Figure 2.**
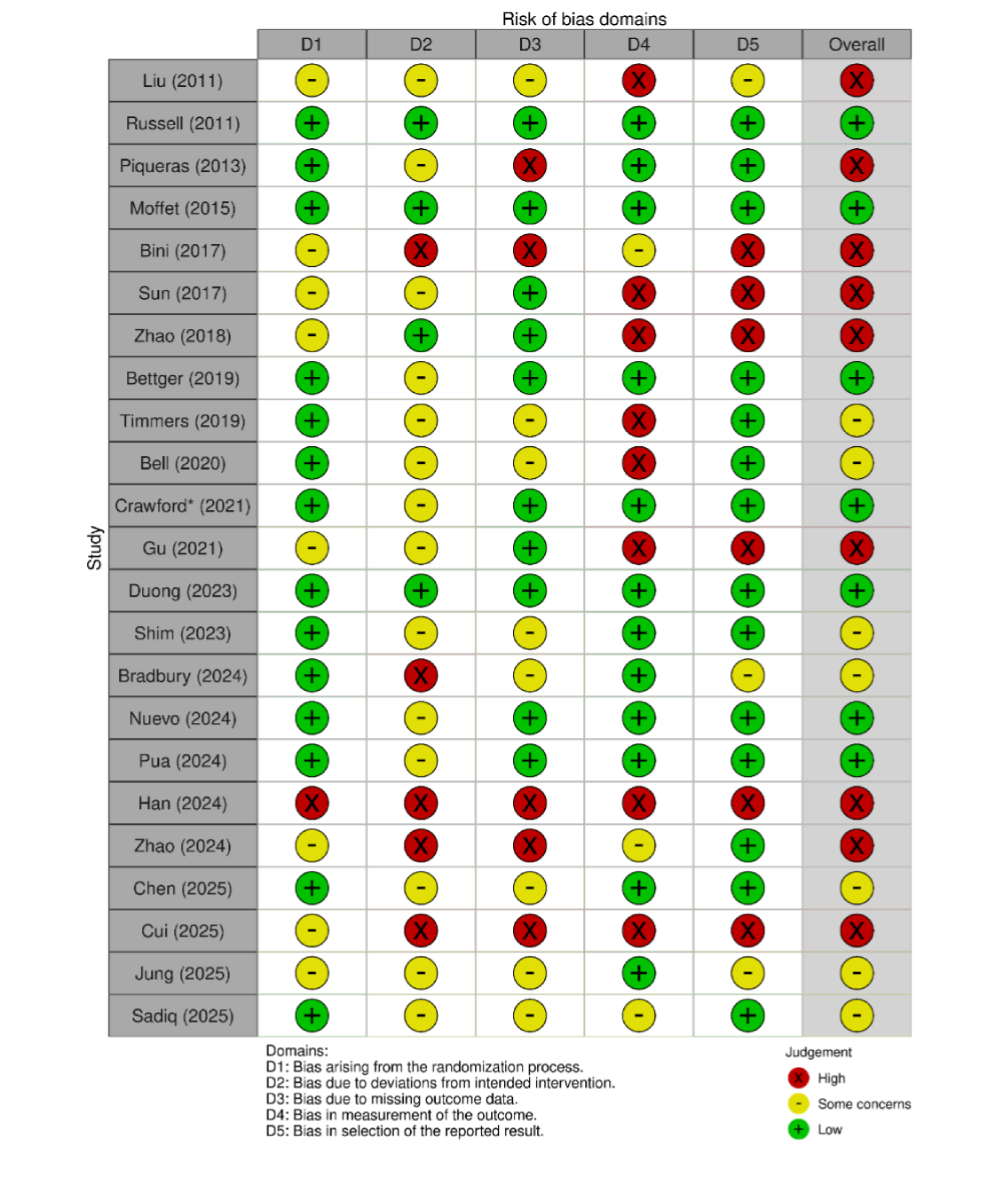
Risk of bias assessment of included randomized controlled trials using the Risk of Bias 2 tool [28-32,46-62].

**Figure 3.**
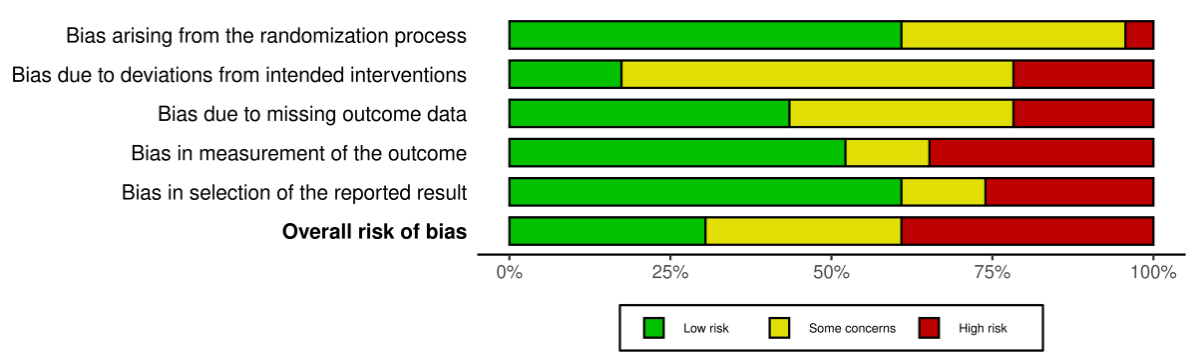
Summary of risk of bias across all included studies using the Cochrane Risk of Bias 2 tool for randomized controlled trials.

Studies classified as having some concerns commonly lacked detailed reporting of allocation concealment, did not clearly specify assessor blinding, or had incomplete information regarding adherence to intended interventions. Studies judged to be at high risk of bias were primarily single-center trials with methodological limitations, including inadequate or unclear randomization procedures, absence of allocation concealment, lack of blinded outcome assessment, and inappropriate handling of missing data. In several cases, interventions were not strictly controlled or involved multiple components, increasing the likelihood of performance bias. The study-level traffic light plot of the domain-level and overall risk-of-bias judgments is presented in Figure 2, and the percentage-based graphical summary of the assessments is shown in Figure 3.

### Primary Outcomes

#### Pain

The MD was used for pain outcomes because all included studies assessed pain using comparable instruments, such as the VAS or NRS, both ranging from 0 to 10. Given the consistency in measurement scale and units, MD allows for direct comparison and preserves clinical interpretability.

**Figure 4.**
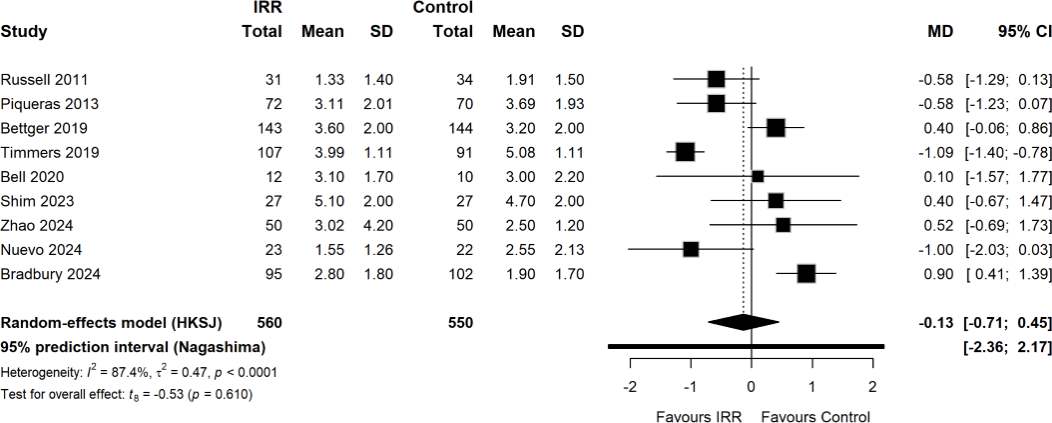
Forest plot of pain scores comparing IRR versus conventional rehabilitation after total knee arthroplasty at short-term follow-up [28,30,32,46,47,52-54,58]. HKSJ: Hartung-Knapp-Sidik-Jonkman; IRR: interactive remote rehabilitation; MD: mean difference.

For short-term pain outcomes, 9 studies involving 1110 participants were included in the meta-analysis [28,30,32,46,47,52-54,58]. Using the HKSJ, no statistically significant difference was observed between groups (MD=−0.13, 95% CI −0.71 to 0.45; *P*=.61). Substantial heterogeneity was detected (*I*^2^=87.4%; τ^2^=0.47). The Nagashima-corrected 95% PI ranged from −2.36 to 2.17 (Figure 4). Sensitivity analysis excluding 2 studies with high risk of bias and included 7 studies involving 868 participants. The pooled effect remained nonsignificant (MD=−0.13, 95% CI −0.88 to 0.61; *P*=.68), with persistently high heterogeneity (*I*^2^=90.2%). The Nagashima-corrected 95% PI (−2.82 to 2.55) indicating that the results were robust to exclusion of the high-risk study (Figure S1 in Multimedia Appendix 1).

**Figure 5.**
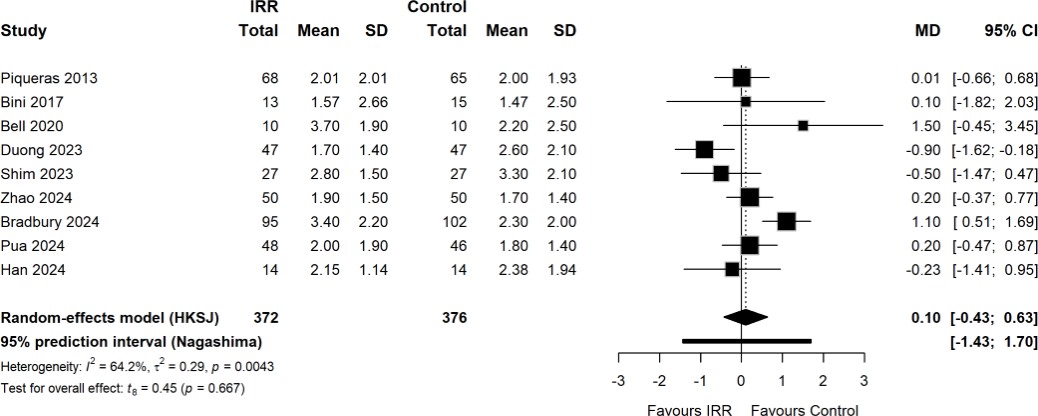
Forest plot of pain scores comparing IRR versus conventional rehabilitation after total knee arthroplasty at mid-term follow-up [28,29,31,32,47,50,53,57,58]. HKSJ: Hartung-Knapp-Sidik-Jonkman; IRR: interactive remote rehabilitation; MD: mean difference.

For mid-term pain outcomes, 9 studies involving 748 participants were included in the meta-analysis [28,29,31,32,47,50,53,57,58]. Using the HKSJ random-effects model, no statistically significant difference was observed between groups (MD=0.10, 95% CI −0.43 to 0.63; *P*=.67). Moderate heterogeneity was observed (*I*^2^=64.2%; τ^2^=0.29). The Nagashima-corrected 95% PI ranged from −1.43 to 1.70 (Figure 5). Sensitivity analysis excluding 4 studies with high risk of bias and included 5 studies involving 459 participants. The pooled effect remained nonsignificant (MD=0.17, 95% CI −1.03 to 1.37; *P*=.71), with increased heterogeneity (*I*^2^=81.5%). The Nagashima-corrected 95% PI (−2.79 to 3.21) also crossed the null value (Figure S2 in Multimedia Appendix 1).

**Figure 6.**
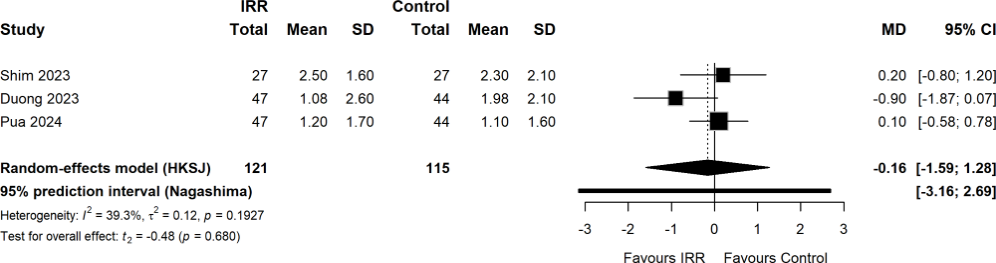
Forest plot of pain scores comparing IRR versus conventional rehabilitation after total knee arthroplasty at long-term follow-up [31,57,58]. HKSJ: Hartung-Knapp-Sidik-Jonkman; IRR: interactive remote rehabilitation; MD: mean difference.

For long-term pain outcomes, 3 studies involving 236 participants were included in the meta-analysis [31,57,58]. No statistically significant difference was observed between groups (MD=−0.16, 95% CI −1.59 to 1.28; *P*=.68). Low-to-moderate heterogeneity was observed (*I*^2^=39.3%; τ^2^=0.119). The Nagashima-corrected 95% PI ranged from −3.16 to 2.69 (Figure 6).

#### WOMAC

The SMD was used for WOMAC outcomes because the included studies reported WOMAC scores using different formats and scales, which precluded direct comparison of absolute values.

**Figure 7.**
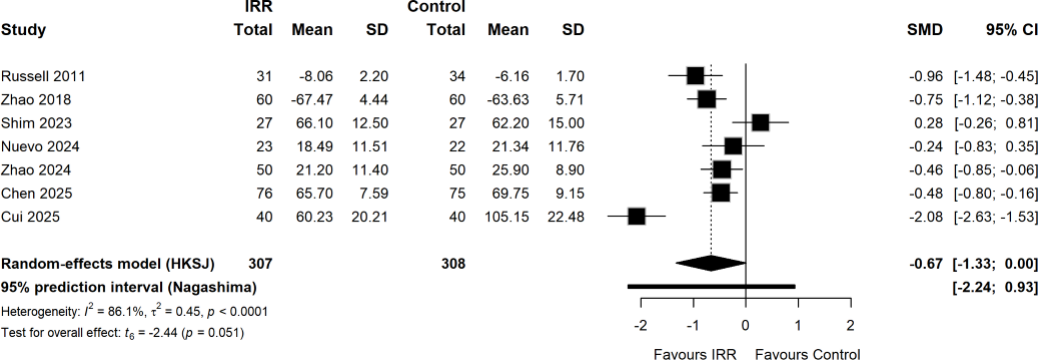
Forest plot of WOMAC scores comparing IRR versus conventional rehabilitation after total knee arthroplasty at short-term follow-up [30,32,46,51,58,59,62]. HKSJ: Hartung-Knapp-Sidik-Jonkman; IRR: interactive remote rehabilitation; SMD: standardized mean difference; WOMAC: Western Ontario and McMaster Universities Osteoarthritis Index.

For short-term WOMAC outcomes, 7 studies [30,32,46,51,58,59,62] involving 615 participants were included. Using the HKSJ random-effects model, no statistically significant difference was observed between groups (SMD=−0.67, 95% CI −1.33 to 0.00; *P*=.05). Substantial heterogeneity was detected (*I*^2^=86.1%; τ^2^=0.46). The Nagashima-corrected 95% PI ranged from −2.24 to 0.93 (Figure 7). Sensitivity analysis excluding studies with an overall high risk of bias and included 4 studies involving 315 participants. The pooled effect remained nonsignificant (SMD=−0.36, 95% CI −1.17 to 0.44; *P*=.25), with substantial heterogeneity (*I*^2^=73.2%). The Nagashima-corrected 95% PI ranged from −2.05 to 1.33 (Figure S3 in Multimedia Appendix 1).

**Figure 8.**
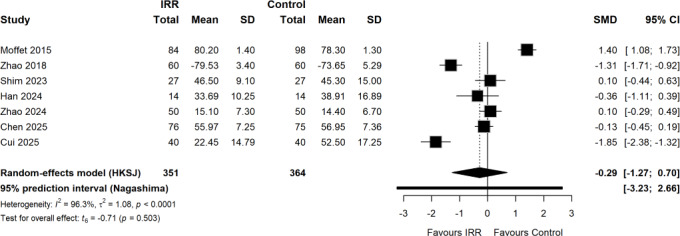
Forest plot of WOMAC scores comparing IRR versus conventional rehabilitation after total knee arthroplasty at mid-term follow-up [29,32,48,51,58,59,62]. HKSJ: Hartung-Knapp-Sidik-Jonkman; IRR: interactive remote rehabilitation; SMD: standardized mean difference; WOMAC: Western Ontario and McMaster Universities Osteoarthritis Index.

For mid-term WOMAC outcomes, 7 studies [29,32,48,51,58,59,62] involving 715 participants were included in the meta-analysis. No statistically significant difference was observed between groups (SMD=−0.29, 95% CI −1.27 to 0.70; *P*=.50). High heterogeneity was detected (*I*^2^=96.3%; τ^2^=1.08). The Nagashima-corrected 95% PI ranged from −3.23 to 2.66 (Figure 8). Sensitivity analysis excluding 4 studies with an overall high risk of bias and included 3 studies involving 387 participants. The pooled effect remained nonsignificant (SMD=0.46, 95% CI −1.61 to 2.54; *P*=.44), with persistently high heterogeneity (*I*^2^=95.7%). The Nagashima-corrected 95% PI ranged from −4.40 to 5.16 (Figure S4 in Multimedia Appendix 1).

**Figure 9.**
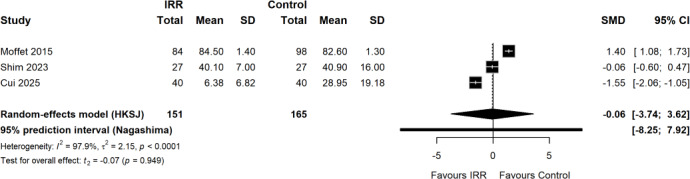
Forest plot of WOMAC scores comparing IRR versus conventional rehabilitation after total knee arthroplasty at long-term follow-up [48,58,62]. HKSJ: Hartung-Knapp-Sidik-Jonkman; IRR: interactive remote rehabilitation; SMD: standardized mean difference; WOMAC: Western Ontario and McMaster Universities Osteoarthritis Index.

For long-term WOMAC outcomes, 3 studies [48,58,62] involving 316 participants were included in the meta-analysis. No statistically significant difference was observed between groups (SMD=−0.06, 95% CI −3.74 to 3.62; *P*=.95). High heterogeneity was observed (*I*^2^=97.9%; τ^2^=2.15). The Nagashima-corrected 95% PI ranged from −8.25 to 7.92 (Figure 9). Sensitivity analysis excluding 1 study with high risk of bias and included 2 studies involving 236 participants. The pooled effect remained nonsignificant (SMD=0.69, 95% CI −8.64 to 10.01; *P*=.52), with persistently high heterogeneity (*I*^2^=95.3%). The Nagashima-corrected 95% PI (−15.19 to 17.51) was wide (Figure S5 in Multimedia Appendix 1).

#### KOOS

The MD was used for KOOS outcomes, as all included studies reported KOOS total scores using the same scale (0‐100), allowing direct comparison of absolute differences between groups.

**Figure 10.**
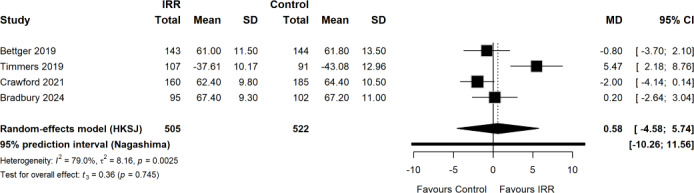
Forest plot of KOOS scores comparing IRR versus conventional rehabilitation after total knee arthroplasty at short-term follow-up [28,52,54,56]. HKSJ: Hartung-Knapp-Sidik-Jonkman; IRR: interactive remote rehabilitation; KOOS: Knee Injury and Osteoarthritis Outcome Score; MD: mean difference.

For short-term KOOS outcomes, 4 studies involving 1027 participants were included in the meta-analysis [28,52,54,56]. No statistically significant difference was observed between groups (MD=0.58, 95% CI −4.58 to 5.74; *P*=.75). Moderate-to-high heterogeneity was observed (*I*^2^=79.0%; τ^2^=8.16). The Nagashima-corrected 95% PI ranged from −10.26 to 11.56 (Figure 10). No sensitivity analysis based on risk of bias was performed, as none of the included studies were judged to be at high risk of bias.

**Figure 11.**
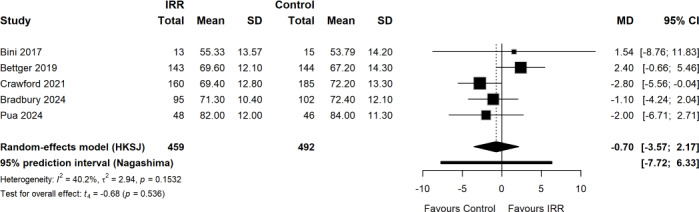
Forest plot of KOOS scores comparing IRR versus conventional rehabilitation after total knee arthroplasty at mid-term follow-up [28,31,50,54,56]. HKSJ: Hartung-Knapp-Sidik-Jonkman; IRR: interactive remote rehabilitation; KOOS: Knee Injury and Osteoarthritis Outcome Score; MD: mean difference.

For mid-term KOOS outcomes, 5 studies involving 951 participants were included in the meta-analysis [28,31,50,54,56]. No statistically significant difference was observed between groups (MD=−0.70, 95% CI −3.57 to 2.17; *P*=.54). Moderate heterogeneity was observed (*I*^2^=40.2%; τ^2^=2.94). The Nagashima-corrected 95% PI ranged from −7.72 to 6.33 (Figure 11). Sensitivity analysis excluding 1 study with high risk of bias and included 4 studies involving 923 participants(n=923). The pooled effect remained nonsignificant (MD=−0.81, 95% CI −4.58 to 2.97; *P*=.55), with slightly increased heterogeneity (*I*^2^=53.8%). The Nagashima-corrected 95% PI ranged from −9.24 to 7.57 (Figure S6 in Multimedia Appendix 1).

**Figure 12.**
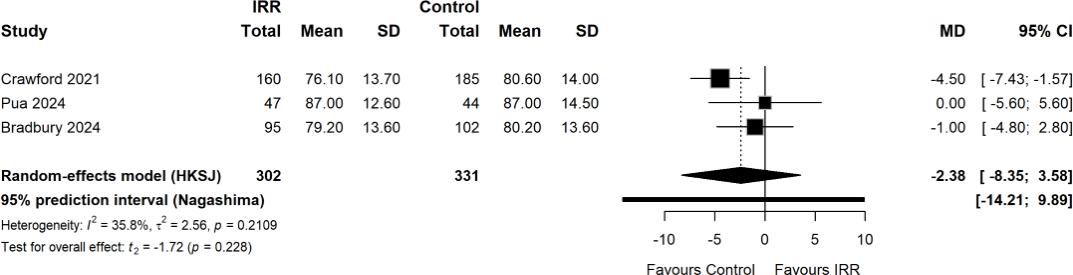
Forest plot of KOOS scores comparing IRR versus conventional rehabilitation after total knee arthroplasty at long-term follow-up [28,31,56]. HKSJ: Hartung-Knapp-Sidik-Jonkman; IRR: interactive remote rehabilitation; KOOS: Knee Injury and Osteoarthritis Outcome Score; MD: mean difference.

For long-term KOOS outcomes, 3 studies involving 633 participants were included in the meta-analysis [28,31,56]. No statistically significant difference was observed between groups (MD=−2.38, 95% CI −8.35 to 3.58; *P*=.23). Low-to-moderate heterogeneity was observed (*I*^2^=35.8%; τ^2^=2.56). The Nagashima-corrected 95% PI ranged from −14.21 to 9.89 (Figure 12).

### Secondary Outcomes

#### ROM

In total, 10 studies were included in the meta-analysis of ROM [28,30-32,46-48,53,54,62]. Overall, no consistent benefit of IRR was observed across active flexion or active extension outcomes at short-, mid-, or long-term follow-up (Figures S7-S18 in Multimedia Appendix 1). Among the 6 primary ROM analyses, only short-term active extension showed a statistically significant but very small difference favoring IRR (MD=0.26, 95% CI 0.04-0.48; *P*=.03; *I*^2^=0%), with a Nagashima-corrected 95% PI of 0.00-0.52. This finding remained statistically significant after exclusion of 2 high-risk studies (MD=0.34, 95% CI 0.09 to 0.59; *P*=.02; *I*^2^=0%), with a Nagashima-corrected 95% PI of 0.03-0.64. Sensitivity analysis for long-term active extension also yielded a marginally significant result (MD=−0.10, 95% CI −0.19 to −0.01; *P*=.046; *I*^2^=0%), although the Nagashima-corrected 95% PI crossed the null value (−0.92 to 0.65). No other primary or sensitivity analyses for ROM outcomes showed statistically significant differences between groups.

#### Quadriceps Muscle Strength

Quadriceps muscle strength was analyzed using SMD because different measurement methods and units were used across studies. In total, 6 studies involving 468 participants were included [30,31,46,47,55,58]. For quadriceps muscle strength, a statistically significant short-term improvement favoring IRR was observed in the primary analysis of 4 studies involving 306 participants (SMD=0.60, 95% CI 0.01-1.19; *P*=.04; *I*^2^=52.3%). However, the Nagashima-corrected 95% PI crossed the null value (−0.63 to 1.87), and the effect was no longer significant after exclusion of 1 high-risk study (SMD=0.71, 95% CI −0.37 to 1.79; *P*=.11). No significant differences were found at mid- or long-term follow-up, and corresponding sensitivity analyses did not materially alter these findings (Figures S19-S24 in Multimedia Appendix 1).

#### TUG

For TUG, only short-term data were available for meta-analysis due to the limited number of studies at longer follow-up periods. In total, 6 studies involving 816 participants were included [28,30,46,47,53,56]. No statistically significant difference was observed between IRR and control (MD=−3.04, 95% CI −8.06 to 1.98; *P*=.18), with substantial heterogeneity (*I*^2^=91%). The Nagashima-corrected 95% PI also crossed the null value (−14.90 to 8.95). Sensitivity analysis excluding 1 high-risk study yielded a similar null finding (MD=−0.58, 95% CI −1.59 to 0.43; *P*=.19; *I*^2^=0%; Figures S25 and S26 in Multimedia Appendix 1).

#### EQ-5D

In total, 5 studies were included in the meta-analysis of EQ-5D [30,52,56,58,60]. No statistically significant differences between IRR and control were observed at short-term follow-up (5 studies, 453 participants; SMD=0.39, 95% CI −0.27 to 1.05; *P*=.17), mid-term follow-up (2 studies, 103 participants; SMD=1.49, 95% CI −13.69 to 16.67; *P*=.43), or long-term follow-up (3 studies, 205 participants; SMD=0.37, 95% CI −3.18 to 3.92; *P*=.70). Heterogeneity was substantial across all time points, and all Nagashima-corrected 95% PIs crossed the null value, indicating considerable uncertainty in the estimated effects (Figures S27-S29 in Multimedia Appendix 1).

#### SF-36

In total, 3 studies reported SF-36 outcomes across multiple domains, with most domains showing higher scores in the intervention group, particularly for physical functioning. However, all included studies were rated as high risk of bias, and results varied across domains. Due to the small number of studies and heterogeneity in outcome domains and follow-up time points, a meta-analysis was not performed, and findings should be interpreted with caution.

#### 6MWT

In total, 2 studies reported 6MWT outcomes at different follow-up time points. Both studies showed no statistically significant differences between groups. Due to the limited number of studies, heterogeneity in follow-up timing, and differences in sample sizes, a meta-analysis was not performed.

### Publication Bias and Additional Analysis

Publication bias (funnel plot and Egger test) and subgroup analyses were not performed due to the limited number of studies available for each outcome, which precluded reliable statistical assessment.

### GRADE

Across outcomes, 95% CIs were used to interpret the uncertainty around the pooled average effects, whereas 95% PIs were used to assess the potential range of true effects across comparable clinical settings. For several outcomes, the CIs indicated no statistically significant average effect, while the PIs were wide and crossed the null value, indicating that effects may differ across settings. The GRADE summary of findings table for the key outcomes is presented in Table 2, and the detailed GRADE evidence profile is provided in Multimedia Appendix 2. The certainty of evidence was generally low to very low across most outcomes, primarily due to substantial heterogeneity, wide CIs and PIs, and risk of bias in several studies. Accordingly, the pooled findings should be interpreted as uncertain estimates rather than definitive evidence of equivalence or superiority.

**Table 2.** GRADE^a^ summary of findings for interactive remote rehabilitation compared with conventional rehabilitation after total knee arthroplasty^b,c^.

Outcomes	Anticipated absolute effects^d^ (95% CI)		Relative effect (95% CI)	Participants (studies), n	Certainty of the evidence (GRADE)	Comments
	Risk with conventional rehabilitation	Risk with interactive remote rehabilitation				
Pain (short-term)	—^e^	MD^f^ 0.13 points lower (0.71 lower to 0.45 higher)	—	1110 (9 RCTs^g^)	⨁⨁◯◯ Low^h,i^	—
WOMAC^j^ (short-term)	—	SMD^k^ 0.67 SD lower (1.33 lower to 0)	—	615 (7 RCTs)	⨁◯◯◯ Very low^h,i,l^	—
KOOS^m^ (short-term)	—	MD 0.58 points higher (4.58 lower to 5.74 higher)	—	1027 (4 RCTs)	⨁⨁◯◯ Low^h,i^	—
Active extension ROM^n^ (short-term)	—	MD 0.26 degrees higher (0.04 higher to 0.48 higher)	—	838 (7 RCTs)	⨁⨁◯◯ Low^l,o^	—
Quadriceps muscle strength (short-term)	—	SMD 0.6 SD higher (0.01 higher to 1.19 higher)	—	306 (4 RCTs)	⨁⨁◯◯ Low^i,l^	—
TUG^p^ (short-term)	—	MD 3.04 seconds lower (8.06 lower to 1.98 higher)	—	816 (6 RCTs)	⨁◯◯◯ Very low^i,l,q^	—
EQ-5D (short-term)	—	SMD 0.39 SD higher (0.27 lower to 1.05 higher)	—	453 (5 RCTs)	⨁◯◯◯ Very low^h,i,l^	—

a GRADE: Grading of Recommendations Assessment, Development and Evaluation.

b GRADE Working Group grades of evidence. High certainty: we are very confident that the true effect lies close to that of the estimate of the effect. Moderate certainty: we are moderately confident in the effect estimate: the true effect is likely to be close to the estimate of the effect, but there is a possibility that it is substantially different. Low certainty: our confidence in the effect estimate is limited: the true effect may be substantially different from the estimate of the effect. Very low certainty: we have very little confidence in the effect estimate: the true effect is likely to be substantially different from the estimate of effect.

c Patient or population: adults undergoing total knee arthroplasty. Setting: postoperative rehabilitation settings after total knee arthroplasty. Intervention: interactive remote rehabilitation. Comparison: conventional rehabilitation.

d The risk in the intervention group (and its 95% CI) is based on the assumed risk in the comparison group and the relative effect of the intervention (and its 95% CI).

e Not available.

f MD: mean difference.

g RCT: randomized controlled trial.

h Downgraded for inconsistency because substantial heterogeneity was observed across studies.

i Downgraded for imprecision because the 95% CI crossed or approached the null value, or because the prediction interval was wide.

j WOMAC: Western Ontario and McMaster Universities Osteoarthritis Index.

k SMD: standardized mean difference.

l Downgraded for risk of bias because some included studies were judged to have a high risk of bias or raised some concerns in the Risk of Bias 2 assessment.

m KOOS: Knee Injury and Osteoarthritis Outcome Score.

n ROM: range of motion.

o Downgraded for imprecision because the effect size was small and its clinical importance was uncertain.

p TUG: Timed Up and Go test.

q Downgraded for very serious inconsistency because heterogeneity was very high and could not be adequately explained.

## Discussion

### Principal Findings

This systematic review and meta-analysis of 23 RCTs involving 2607 participants evaluated the effectiveness of IRR compared with conventional rehabilitation after TKA. Across the prespecified primary outcomes, IRR did not demonstrate statistically significant advantages over conventional rehabilitation for pain or patient-reported function, including WOMAC and KOOS outcomes, at short-, mid-, or long-term follow-up. Findings for most secondary outcomes were similarly neutral, with no consistent benefits observed for mobility or health-related quality of life. Among ROM outcomes, only a very small improvement in short-term active extension favored IRR, and a marginal signal for long-term active extension emerged only in sensitivity analysis; however, the magnitude and consistency of these findings were limited. A short-term improvement in quadriceps muscle strength was also observed in the primary analysis, but this effect was not robust after exclusion of studies at high risk of bias and was not maintained at later follow-up. Overall, the evidence does not indicate a consistent average benefit of IRR over conventional rehabilitation after TKA based on the pooled effects and their 95% CIs. In addition, wide PIs in several analyses suggest that the true effects of IRR may vary across different clinical settings. These findings should therefore be interpreted cautiously considering heterogeneity, risk of bias in several included studies, and the generally low to very low certainty of evidence according to GRADE.

### Interpretation and Comparison With Previous Literature

Compared with previous meta-analyses, which primarily evaluated remote rehabilitation as a broad or technology-based category, this study focused specifically on IRR defined by bidirectional communication [14-16,18,64,65]. Many of these syntheses were published relatively early, when the available randomized evidence was still limited, and consequently included a smaller number of trials drawn largely from earlier generations of remote rehabilitation [14-16,18,64,65]. In contrast, this review incorporates a substantially updated evidence base. Among the 23 RCTs included in our analysis, 9 were published in 2024 or later and, to our knowledge, are synthesized in a meta-analysis of post-TKA IRR for the first time. This expanded body of recent evidence provides a more contemporary assessment of the effectiveness of IRR in the context of rapidly evolving digital rehabilitation technologies and care models. The earliest meta-analysis included only 4 RCTs and reported that telerehabilitation achieved comparable pain relief and greater improvements than face-to-face rehabilitation [18]. However, its conclusions were based on a limited evidence base, and one included trial used home-based rehabilitation monitored only by periodic telephone calls, which does not clearly meet the criteria for telerehabilitation or the IRR definition used in our review [66]. This may have contributed to conceptual heterogeneity and overestimation of benefit. Jiang et al [64] included 4 early RCTs and concluded that in-home telerehabilitation achieved pain control comparable to face-to-face rehabilitation and might provide better functional recovery after TKA, while also acknowledging the need for larger samples to confirm its efficacy. Tsang et al [16] included 11 RCTs and reported that telerehabilitation achieved pain and functional outcomes comparable to conventional in-person rehabilitation. Although most included interventions were consistent with remote rehabilitation, several trials involving telephone-based follow-up or home-based rehabilitation programs were also synthesized within the same evidence base [66-68]. Zhang et al [14] compared home-based telerehabilitation with hospital-based rehabilitation, focusing primarily on differences in rehabilitation setting rather than on the interactive characteristics of remote care. Another recent review focused on the needs and experiences of patients participating in remote rehabilitation rather than clinical effectiveness [65]. Moreover, it synthesized qualitative evidence from both total hip arthroplasty and TKA, making its scope and research question fundamentally different from the present TKA-specific efficacy review [65]. The most recent meta-analysis included a larger body of RCTs and suggested that remote rehabilitation may outperform face-to-face rehabilitation for selected outcomes after TKA [15]. However, it still synthesized some home-based rehabilitation interventions, did not treat interactivity as a defining feature, and pooled outcomes across different follow-up periods, which may have obscured time-dependent rehabilitation effects [15]. These interventions differ substantially from feedback-enabled IRR and may have introduced conceptual heterogeneity. Accordingly, this review adopted bidirectional patient-provider interaction as a required inclusion criterion to provide a more focused assessment of IRR after TKA.

While IRR has shown potential benefits in other clinical contexts, including geriatric postural control and neurological rehabilitation, our synthesis did not demonstrate consistent superiority over conventional rehabilitation following TKA [69-72]. These results suggest that interactivity alone may not suffice to produce uniform improvements in outcomes, and that effects likely depend on intervention design, intensity, and patient characteristics [73]. Compared with prior reviews, this approach reduces conceptual heterogeneity and provides a clearer assessment of what IRR contributes after TKA.

Taken together, these comparisons highlight the distinct contribution of this review. By focusing specifically on bidirectional interactive interventions, incorporating time-stratified analyses, and applying conservative meta-analytic methods, including HKSJ CIs and Nagashima-corrected PIs, this study provides a more focused and clinically interpretable assessment of IRR after TKA. The findings suggest that IRR may be a feasible model for extending postoperative rehabilitation support, particularly where access to in-person services is constrained, but they do not establish superiority over conventional rehabilitation.

### Outcome-Specific Findings

The outcome-specific findings should be interpreted in the context of the overall absence of a consistent advantage of IRR over conventional rehabilitation. Across the primary outcomes, pain and patient-reported function, assessed using WOMAC and KOOS, did not show stable benefits favoring IRR at any follow-up period. These findings suggest that, although interactive technologies may facilitate rehabilitation delivery, they do not necessarily translate into superior symptom relief or broader self-reported functional recovery compared with established postoperative rehabilitation approaches [74].

Several secondary outcomes showed isolated signals that warrant cautious interpretation. A small short-term difference in active extension ROM favored IRR, but the magnitude of this effect was very limited and is unlikely to indicate a clinically meaningful improvement on its own. Similarly, quadriceps muscle strength showed a short-term advantage in the primary analysis, but this finding was no longer robust after exclusion of studies at high risk of bias and was not maintained at later follow-up. Mobility assessed by TUG and health-related quality of life assessed by EQ-5D did not demonstrate significant advantages for IRR, while evidence for SF-36 and 6MWT remained insufficient for firm quantitative conclusions. Taken together, these patterns suggest that IRR may produce modest benefits in selected domains of early postoperative recovery, but the current evidence does not support a consistent or durable effect across the broader spectrum of TKA rehabilitation outcomes.

### Clinical Meaning of IRR Despite Uncertain Superiority

The interpretation of these findings should also consider the nature of the comparator interventions. The relative effectiveness of IRR may depend not only on the interactive features of the remote program itself but also on the intensity and quality of the rehabilitation model against which it is compared [75-77]. A recent meta-analysis of pulmonary telerehabilitation similarly argued that remote rehabilitation was more likely to show favorable effects when compared with lower-intensity programs or usual care, whereas apparent advantages were attenuated when the comparator was high-intensity, face-to-face supervised rehabilitation [78]. This perspective is relevant to this review, in which control conditions range from conventional in-person rehabilitation to outpatient care, usual care, and unsupervised home exercise. Accordingly, the absence of consistent superiority for IRR should be interpreted as context-dependent rather than as evidence of universally equivalent or ineffective remote rehabilitation [79].

Importantly, the absence of demonstrated superiority should not be interpreted as evidence that IRR lacks clinical value [80]. The potential contribution of IRR may lie not only in producing better outcomes than conventional rehabilitation but also in extending access to structured postoperative support, maintaining continuity of care after discharge, and enabling remote monitoring, feedback, and communication between patients and rehabilitation professionals [80,81]. These features may be particularly relevant in contexts where in-person rehabilitation is difficult to access or sustain [82].

However, the present findings indicate that the availability of interactive technology alone is insufficient to guarantee better clinical outcomes. Whether IRR translates into meaningful clinical benefit likely depends on how interaction is operationalized, including the intensity and timing of feedback, the degree of personalization, the integration of monitoring data into clinical decision-making, and the characteristics of the patients receiving the intervention [22,73]. Emerging technologies may further reshape these interactive mechanisms [74,83]. For example, high-speed and low-latency communication infrastructure, such as 5G-enabled systems, may support more stable real-time supervision and data transmission, while AI may facilitate automated movement assessment, adaptive exercise progression, and more individualized feedback within remote rehabilitation platforms [84,85]. Future trials should therefore evaluate not only whether IRR is effective but also which technologically enabled interactive components meaningfully improve postoperative recovery after TKA [86].

### Implications for Practice and Future Research

This broader perspective is aligned with recent health systems evidence on rehabilitation delivery arrangements. A 2026 Cochrane overview concluded that the evidence for telerehabilitation and related rehabilitation service models remains limited and is often of low certainty and highlighted the need for future studies to evaluate organizational, implementation, and equity-related outcomes in addition to patient-level clinical effects [87]. Accordingly, IRR after TKA should be considered not only as a therapeutic intervention but also as a potentially important service-delivery model whose value may extend to continuity, accessibility, and organization of postoperative rehabilitation [88]. Face-to-face rehabilitation may remain preferable for patients who require close hands-on supervision, complex clinical assessment, or more intensive multidisciplinary support [2,13]. From a practical perspective, IRR may be particularly relevant for patients who face geographic, mobility, scheduling, or resource-related barriers to repeated facility-based rehabilitation, as it can extend structured postoperative support beyond the clinic and maintain communication with rehabilitation professionals [24,89].

Future research should therefore move beyond broad labels and adopt standardized, transparent definitions of therapeutic interactivity [90]. Trials should clearly report the specific interactive components being tested, including whether communication is synchronous, asynchronous, or hybrid; the frequency, timing, and intensity of clinician feedback; the use of monitoring data to adjust exercise prescriptions; the degree of personalization; and strategies used to support adherence [13,90,91]. In addition, subsequent studies should determine which patient groups are most likely to benefit from IRR, rather than assuming a uniform effect across all individuals undergoing TKA [13,87,91]. Beyond conventional clinical outcomes, future research should more systematically assess adherence, patient experience, satisfaction, cost-effectiveness, health care use, implementation feasibility, and equity of access [91]. Such evidence will be essential for defining the appropriate role of IRR within post-TKA care pathways and for identifying the interactive models, patient populations, and service contexts in which remote rehabilitation is most likely to provide meaningful value.

### Limitations

This study has several limitations. First, substantial heterogeneity persisted across studies in intervention characteristics, including duration, frequency, and technological platforms. Second, the number of trials for several outcomes was limited, reducing the precision and interpretability of pooled estimates. In addition, the *I*^2^ statistic may be biased when the number of included studies is small and should therefore be interpreted with caution [92]. Third, several included studies were judged to have high risk of bias, and blinding of participants and therapists was generally not feasible, which may have influenced subjective outcome reporting. Fourth, wide PIs were observed in several analyses, indicating considerable uncertainty in the range of true effects that might be expected across different clinical settings. Finally, subgroup analyses and small-study effect assessments were not performed due to the limited number of studies for most outcomes, and cost-effectiveness and patient-reported satisfaction were not quantitatively evaluated.

### Conclusions

This review is innovative in focusing specifically on IRR defined by bidirectional patient-provider communication, rather than treating all remote or technology-assisted rehabilitation as a single intervention category. In contrast to previous reviews, it combines this more precise conceptual framework with time-stratified outcome synthesis and conservative random-effects inference using the HKSJ method and PIs. By doing so, this study provides a clearer and more clinically interpretable assessment of the current evidence: in light of substantial heterogeneity, risk of bias, and generally low to very low certainty of evidence, IRR has not demonstrated consistent superiority over conventional rehabilitation after TKA, although it may serve as a feasible care model in settings where access to in-person rehabilitation is limited. These findings contribute to the digital rehabilitation field by clarifying both the promise and the current evidentiary limits of IRR, and they may inform clinical service planning as well as the design of future trials aimed at identifying which interactive features, patient groups, and implementation contexts are most likely to benefit.

## Supplementary material

10.2196/89321 Multimedia Appendix 1 Forest and sensitivity analysis.

10.2196/89321 Multimedia Appendix 2 Grading of Recommendations Assessment, Development and Evaluation evidence profile for key outcomes comparing interactive remote rehabilitation with conventional rehabilitation after total knee arthroplasty.

10.2196/89321 Checklist 1 PRISMA 2020 checklist.

10.2196/89321 Checklist 2 PRISMA-S checklist.

10.2196/89321 Checklist 3 PRISMA-S search reporting details and complete database-specific search strategies.

## Abbreviations

6MWT: 6-minute walk test
GRADE: Grading of Recommendations Assessment, Development and Evaluation
HKSJ: Hartung-Knapp-Sidik-Jonkman
IRR: interactive remote rehabilitation
KOOS: Knee Injury and Osteoarthritis Outcome Score
MD: mean difference
NRS: numeric rating scale
PI: prediction interval
PRISMA: Preferred Reporting Items for Systematic Reviews and Meta-Analyses
PRISMA-S: Preferred Reporting Items for Systematic Reviews and Meta-Analyses Literature Search Extension
RCT: randomized controlled trial
ROM: range of motion
SF-36: 36-Item Short Form Survey
SMD: standardized mean difference
TKA: total knee arthroplasty
TUG: Timed Up and Go test
VAS: visual analog scale
WOMAC: Western Ontario and McMaster Universities Osteoarthritis Index
