# Supplementary material for: Effectiveness of Interactive Remote Rehabilitation After Total Knee Arthroplasty: Systematic Review and Meta-Analysis of Randomized Controlled Trials
Source: J Med Internet Res. 2026 Aug 7;28:e89321. doi: 10.2196/89321 (PMC13449792; doi:10.2196/89321)
Supplement: Supplementary file 1 [file jmir-v28-e89321-s004.pdf]

PRISMA-S Checklist

Section/topic	#	Checklist item	Location(s) Reported
**INFORMATION SOURCES AND METHODS**			
Database name	1	Name each individual database searched, stating the platform for each.	Line 229-233
Multi-database searching	2	If databases were searched simultaneously on a single platform, state the name of the platform, listing all of the databases searched.	Not applicable
Study registries	3	List any study registries searched.	PROSPERO (CRD420251049015)<br>Line 210
Online resources and browsing	4	Describe any online or print source purposefully searched or browsed (e.g., tables of contents, print conference proceedings, web sites), and how this was done.	Not applicable
Citation searching	5	Indicate whether cited references or citing references were examined, and describe any methods used for locating cited/citing references (e.g., browsing reference lists, using a citation index, setting up email alerts for references citing included studies).	Reference lists of relevant articles and included studies were manually checked. Formal forward citation searching using citation indexes and citation alerts was not performed.
Contacts	6	Indicate whether additional studies or data were sought by contacting authors, experts, manufacturers, or others.	Not performed
Other methods	7	Describe any additional information sources or search methods used.	No additional information sources or search methods were used beyond the database searches and manual checking of reference lists of relevant articles and included studies.
**SEARCH STRATEGIES**			

Full search strategies	8	Include the search strategies for each database and information source, copied and pasted exactly as run.	Supplementary 2
Limits and restrictions	9	Specify that no limits were used, or describe any limits or restrictions applied to a search (e.g., date or time period, language, study design) and provide justification for their use.	Line 213-227
Search filters	10	Indicate whether published search filters were used (as originally designed or modified), and if so, cite the filter(s) used.	None
Prior work	11	Indicate when search strategies from other literature reviews were adapted or reused for a substantive part or all of the search, citing the previous review(s).	None
Updates	12	Report the methods used to update the search(es) (e.g., rerunning searches, email alerts).	None
Dates of searches	13	For each search strategy, provide the date when the last search occurred.	Line 232<br>May 5, 2026
**PEER REVIEW**			
Peer review	14	Describe any search peer review process.	Line 239
**MANAGING RECORDS**			
Total Records	15	Document the total number of records identified from each database and other information sources.	**Yes**<br>**Figure 1**
Deduplication	16	Describe the processes and any software used to deduplicate records from multiple database searches and other information sources.	Yes

PRISMA-S: An Extension to the PRISMA Statement for Reporting Literature Searches in Systematic Reviews

Rethlefsen ML, Kirtley S, Waffenschmidt S, Ayala AP, Moher D, Page MJ, Koffel JB,
PRISMA-S Group.
Last updated February 27, 2020.
