# Supplementary material for: Effectiveness of Interactive Remote Rehabilitation After Total Knee Arthroplasty: Systematic Review and Meta-Analysis of Randomized Controlled Trials
Source: J Med Internet Res. 2026 Aug 7;28:e89321. doi: 10.2196/89321 (PMC13449792; doi:10.2196/89321)
Supplement: Supplementary file 2 [file jmir-v28-e89321-s001.docx]

**Figure S1.** Sensitivity analysis excluding high-risk studies for short-term pain

**Figure S2.** Sensitivity analysis excluding high-risk studies for mid-term pain

**Figure S3.** Sensitivity analysis excluding high-risk studies for short-term WOMAC

**Figure S4.** Sensitivity analysis excluding high-risk studies for mid-term WOMAC

**Figure S5.** Sensitivity analysis excluding high-risk studies for long-term WOMAC

**Figure S6.** Sensitivity analysis excluding high-risk studies for mid-term KOOS

**Figure S7.** Forest plot of ROM (active flexion) comparing interactive remote rehabilitation versus conventional rehabilitation after TKA at short-term follow-up.

**Figure S8.** Sensitivity analysis excluding high-risk studies for short-term ROM (active flexion).

**Figure S9.** Forest plot of ROM (active flexion) comparing interactive remote rehabilitation versus conventional rehabilitation after TKA at mid-term follow-up.

**Figure S10.** Sensitivity analysis excluding high-risk studies for mid-term ROM (active flexion).

**Figure S11.** Forest plot of ROM (active flexion) comparing interactive remote rehabilitation versus conventional rehabilitation after TKA at long-term follow-up.

**Figure S12.** Sensitivity analysis excluding high-risk studies for long-term ROM (active flexion).

**Figure S13.** Forest plot of ROM (active extension) comparing interactive remote rehabilitation versus conventional rehabilitation after TKA at short-term follow-up.

**Figure S14.** Sensitivity analysis excluding high-risk studies for short-term ROM (active extension).

**Figure S15.** Forest plot of ROM (active extension) comparing interactive remote rehabilitation versus conventional rehabilitation after TKA at mid-term follow-up.

**Figure S16.** Sensitivity analysis excluding high-risk studies for mid-term ROM (active extension).

**Figure S17.** Forest plot of ROM (active extension) comparing interactive remote rehabilitation versus conventional rehabilitation after TKA at long-term follow-up.

**Figure S18.** Sensitivity analysis excluding high-risk studies for long-term ROM (active extension).

**Figure S19.** Forest plot of quadriceps muscle strength comparing interactive remote rehabilitation versus conventional rehabilitation after TKA at short-term follow-up

**Figure S20.** Sensitivity analysis excluding high-risk studies for short-term quadriceps muscle strength

**Figure S21.** Forest plot of quadriceps muscle strength comparing interactive remote rehabilitation versus conventional rehabilitation after TKA at mid-term follow-up

**Figure S22.** Sensitivity analysis excluding high-risk studies for mid-term quadriceps muscle strength

**Figure S23.** Forest plot of quadriceps muscle strength comparing interactive remote rehabilitation versus conventional rehabilitation after TKA at long-term follow-up

**Figure S24.** Sensitivity analysis excluding high-risk studies for long-term quadriceps muscle strength

**Figure S25.** Forest plot of TUG comparing interactive remote rehabilitation versus conventional rehabilitation after TKA at short-term follow-up

**Figure S26.** Sensitivity analysis excluding high-risk studies for short-term TUG

**Figure S27.** Forest plot of EQ-5D comparing interactive remote rehabilitation versus conventional rehabilitation after TKA at short-term follow-up

**Figure S28.** Forest plot of EQ-5D comparing interactive remote rehabilitation versus conventional rehabilitation after TKA at mid-term follow-up

**Figure S29.** Forest plot of EQ-5D comparing interactive remote rehabilitation versus conventional rehabilitation after TKA at long-term follow-up

### References

28. Bradbury TL, McConnell MJ, Whitacre D, Naylor BH, Gibson BT, DeCook CA. A remote physical therapy program demonstrates similar outcomes compared to in-person, supervised physical therapy after same-day discharge total knee arthroplasty: a randomized clinical trial. J Arthroplasty. Nov 2024;39(11):2725-2730. [doi: 10.1016/j.arth.2024.05.040] [Medline: 38768768]

30. Nuevo M, Rodríguez-Rodríguez D, Jauregui R, et al. Telerehabilitation following fast-track total knee arthroplasty is effective and safe: a randomized controlled trial with the ReHub® platform. Disabil Rehabil. Jun 2024;46(12):2629-2639. [doi: 10.1080/09638288.2023.2228689] [Medline: 37403684]

31. Pua YH, Yeo SJ, Clark RA, et al. Cost and outcomes of Hospital-based Usual cAre versus Tele-monitor self-directed Rehabilitation (HUATR) in patients with total knee arthroplasty: a randomized, controlled, non-inferiority trial. Osteoarthr Cartil. May 2024;32(5):601-611. [doi: 10.1016/j.joca.2023.11.017] [Medline: 38049030]

32. Zhao R, Cheng L, Zheng Q, et al. A smartphone application-based remote rehabilitation system for post-total knee arthroplasty rehabilitation: a randomized controlled trial. J Arthroplasty. Mar 2024;39(3):575-581. [doi: 10.1016/j.arth.2023.08.019] [Medline: 37572720]

46. Russell TG, Buttrum P, Wootton R, Jull GA. Internet-based outpatient telerehabilitation for patients following total knee arthroplasty: a randomized controlled trial. J Bone Joint Surg Am. Jan 19, 2011;93(2):113-120. [doi: 10.2106/JBJS.I.01375] [Medline: 21248209]

47. Piqueras M, Marco E, Coll M, et al. Effectiveness of an interactive virtual telerehabilitation system in patients after total knee arthoplasty: a randomized controlled trial. J Rehabil Med. Apr 2013;45(4):392-396. [doi: 10.2340/16501977-1119] [Medline: 23474735]

48. Moffet H, Tousignant M, Nadeau S, et al. In-home telerehabilitation compared with face-to-face rehabilitation after total knee arthroplasty: a noninferiority randomized controlled trial. J Bone Joint Surg Am. Jul 15, 2015;97(14):1129-1141. [doi: 10.2106/JBJS.N.01066] [Medline: 26178888]

52. Timmers T, Janssen L, van der Weegen W, et al. The effect of an app for day-to-day postoperative care education on patients with total knee replacement: randomized controlled trial. JMIR Mhealth Uhealth. Oct 21, 2019;7(10):e15323. [doi: 10.2196/15323] [Medline: 31638594]

53. Bell KM, Onyeukwu C, Smith CN, et al. A portable system for remote rehabilitation following a total knee replacement: a pilot randomized controlled clinical study. Sensors (Basel). Oct 27, 2020;20(21):6118. [doi: 10.3390/s20216118] [Medline: 33121204]

54. Prvu Bettger J, Green CL, Holmes DN, et al. Effects of virtual exercise rehabilitation in-home therapy compared with traditional care after total knee arthroplasty: VERITAS, a randomized controlled trial. J Bone Joint Surg Am. Jan 15, 2020;102(2):101-109. [doi: 10.2106/JBJS.19.00695] [Medline: 31743238]

55. Gu SY. Effect of home-based exercise rehabilitation under remote guidance on muscle strength recovery after total knee arthroplasty [Article in Chinese]. J Pract Med Tech. 2021;28(2):221-224. [doi: 10.19522/j.cnki.1671-5098.2021.02.041]

56. Crawford DA, Duwelius PJ, Sneller MA, et al. 2021 Mark Coventry Award: use of a smartphone-based care platform after primary partial and total knee arthroplasty: a prospective randomized controlled trial. Bone Joint J. Jun 2021;103-B(6 Suppl A):3-12. [doi: 10.1302/0301-620X.103B6.BJJ-2020-2352.R1] [Medline: 34053272]

57. Duong V, Robbins SR, Dennis S, Venkatesha V, Ferreira ML, Hunter DJ. Combined digital interventions for pain reduction in patients undergoing knee replacement: a randomized clinical trial. JAMA Netw Open. Sep 5, 2023;6(9):e2333172. [doi: 10.1001/jamanetworkopen.2023.33172] [Medline: 37713201]

58. Shim GY, Kim EH, Lee SJ, et al. Postoperative rehabilitation using a digital healthcare system in patients with total knee arthroplasty: a randomized controlled trial. Arch Orthop Trauma Surg. Oct 2023;143(10):6361-6370. [doi: 10.1007/s00402-023-04894-y] [Medline: 37129691]

59. Chen W, Qin X, Yang H, Tan Z, Liu P. Impact of cloud-based follow-up exercise prescription on knee function and quality of life in homebound patients post-total knee arthroplasty: a randomized controlled trial. J Orthop Surg Res. Aug 30, 2025;20(1):809. [doi: 10.1186/s13018-025-06187-z] [Medline: 40885961]

60. Jung SJ, Kim JH, Rhee SJ. Home-based rehabilitation using wearable motion tracker with smart phone application feedback is as effective as conventional self-directed rehabilitation after total knee arthroplasty: randomized controlled trial. BMC Sports Sci Med Rehabil. Dec 24, 2025;17(1):370. [doi: 10.1186/s13102-025-01374-1] [Medline: 41444603]

61. Sadiq S, Noor R, Akram R. Effect of lifestyle modification through web-based telerehabilitation monitoring combined with supervised sensorimotor training after total knee arthroplasty: randomized controlled trial. JMIR Mhealth Uhealth. Oct 2, 2025;13:e64643. [doi: 10.2196/64643] [Medline: 41037782]

62. Cui S, Chen YE, Zhou LN, et al. Effects of digital intelligence–based remote home intervention on knee joint function and quality of life in patients after total knee arthroplasty [Article in Chinese]. J Nurs Training. 2025;40(23):2525-2532. [doi: 10.16821/j.cnki.hsjx.2025.23.010]
