# Supplementary material for: Effectiveness of Interactive Remote Rehabilitation After Total Knee Arthroplasty: Systematic Review and Meta-Analysis of Randomized Controlled Trials
Source: J Med Internet Res. 2026 Aug 7;28:e89321. doi: 10.2196/89321 (PMC13449792; doi:10.2196/89321)
Supplement: Supplementary file 3 [file jmir-v28-e89321-s002.docx]

| **Interactive remote rehabilitation compared to Conventional rehabilitation for Adults undergoing total knee arthroplasty**  **Bibliography:** | | | | | | | | | | | |
| --- | --- | --- | --- | --- | --- | --- | --- | --- | --- | --- | --- |
| **Certainty assessment** | | | | | | | **Summary of findings** | | | | |
| **Participants (studies) Follow-up** | **Risk of bias** | **Inconsistency** | **Indirectness** | **Imprecision** | **Publication bias** | **Overall certainty of evidence** | **Study event rates (%)** | | **Relative effect (95% CI)** | **Anticipated absolute effects** | |
|  |  |  |  |  |  |  | **With Conventional rehabilitation** | **With Interactive remote rehabilitation** |  | **Risk with Conventional rehabilitation** | **Risk difference with Interactive remote rehabilitation** |
| **Pain** | | | | | | | | | | | |
| 1110 (9 RCTs) | not serious | serious^a^ | not serious | serious^b^ | none | ⨁⨁◯◯ Low^a,b^ | 550 | 560 | - | 550 | MD **0.13 points lower** (0.71 lower to 0.45 higher) |
| **WOMAC** | | | | | | | | | | | |
| 615 (7 RCTs) | serious^c^ | serious^a^ | not serious | serious^b^ | none | ⨁◯◯◯ Very low^a,b,c^ | 308 | 307 | - | - | SMD **0.67 SD lower** (1.33 lower to 0 ) |
| **KOOS** | | | | | | | | | | | |
| 1027 (4 RCTs) | not serious | serious^a^ | not serious | serious^b^ | none | ⨁⨁◯◯ Low^a,b^ | 522 | 505 | - | 522 | MD **0.58 points higher** (4.58 lower to 5.74 higher) |
| **Active extension ROM** | | | | | | | | | | | |
| 838 (7 RCTs) | serious^c^ | not serious | not serious | serious^d^ | none | ⨁⨁◯◯ Low^c,d^ | 422 | 416 | - | 422 | MD **0.26 degrees higher** (0.04 higher to 0.48 higher) |
| **Quadriceps muscle strength** | | | | | | | | | | | |
| 306 (4 RCTs) | serious^c^ | not serious | not serious | serious^b^ | none | ⨁⨁◯◯ Low^b,c^ | 153 | 153 | - | - | SMD **0.6 SD higher** (0.01 higher to 1.19 higher) |
| **TUG** | | | | | | | | | | | |
| 816 (6 RCTs) | serious^c^ | very serious^e^ | not serious | serious^b^ | none | ⨁◯◯◯ Very low^b,c,e^ | 423 | 393 | - | 423 | MD **3.04 seconds lower** (8.06 lower to 1.98 higher) |
| **EQ-5D** | | | | | | | | | | | |
| 453 (5 RCTs) | serious^c^ | serious^a^ | not serious | serious^b^ | none | ⨁◯◯◯ Very low^a,b,c^ | 224 | 229 | - | - | SMD **0.39 SD higher** (0.27 lower to 1.05 higher) |

**CI:** confidence interval; **MD:** mean difference; **SMD:** standardised mean difference

#### Explanations

a. Downgraded for inconsistency because substantial heterogeneity was observed across studies.

b. Downgraded for imprecision because the 95% confidence interval crossed or approached the null value, or because the prediction interval was wide.

c. Downgraded for risk of bias because some included studies were judged to have a high risk of bias or raised some concerns in the RoB 2 assessment.

d. Downgraded for imprecision because the effect size was small and its clinical importance was uncertain.

e. Downgraded for very serious inconsistency because heterogeneity was very high and could not be adequately explained.
