# Supplementary material for: Effectiveness of Interactive Remote Rehabilitation After Total Knee Arthroplasty: Systematic Review and Meta-Analysis of Randomized Controlled Trials
Source: J Med Internet Res. 2026 Aug 7;28:e89321. doi: 10.2196/89321 (PMC13449792; doi:10.2196/89321)
Supplement: Supplementary file 5 [file jmir-v28-e89321-s005.docx]

**Supplementary 2. PRISMA-S Search Reporting Details and Complete Database-Specific Search Strategies**

**1. PRISMA-S Reporting Details**

This supplementary file reports the literature search in accordance with the PRISMA-S extension. The search strategy was developed by two authors and independently checked by two additional authors for completeness, consistency, and relevance to the review question. The search strategy was not formally peer reviewed by an external information specialist.

**1.1 Databases searched:**

PubMed (MEDLINE), Cochrane Central Register of Controlled Trials (CENTRAL), Embase via Ovid, Web of Science Core Collection, CINAHL via EBSCOhost, Scopus, and China National Knowledge Infrastructure (CNKI).

**1.2 Search date:**

All databases were searched from inception to May 5, 2026. The updated search expanded the original search conducted up to May 25, 2025.

**1.3 Search concepts:**

The search combined three main concepts: total knee arthroplasty, remote or digital rehabilitation, and randomized controlled trials. CENTRAL was not restricted by an additional randomized trial filter because CENTRAL primarily indexes controlled trials.

**1.4 Limits:**

No date restriction was applied. Studies published in English or Chinese were eligible because these were the languages that could be reliably assessed by the review team.

**1.5 Supplementary searching:**

Reference lists of included studies and relevant systematic reviews were manually screened.

**1.6 Search methods not performed:**

Grey literature databases, trial registries, and conference proceedings were not searched because this review focused on peer-reviewed randomized controlled trials with extractable outcome data. Citation searching was not performed as a separate search method. No automated search tools or text-mining tools were used.

**1.7 Record management:**

All retrieved records were imported into EndNote 21 and Rayyan. Duplicate records were removed before title and abstract screening.

**Table S1. PRISMA-S Reporting Details for the Literature Search**

| PRISMA-S reporting item | Reporting in this review |
| --- | --- |
| Reporting guideline | The literature search was reported in accordance with the PRISMA-S extension. |
| Search development | The search strategy was developed by two authors and independently checked by two additional authors for completeness, consistency, and relevance to the review question. |
| External peer review | The search strategy was not formally peer reviewed by an external information specialist. |
| Databases searched | PubMed (MEDLINE), CENTRAL, Embase via Ovid, Web of Science Core Collection, CINAHL via EBSCOhost, Scopus, and CNKI. |
| Search date | All databases were searched from inception to May 5, 2026. |
| Search update | The updated search expanded the original search conducted up to May 25, 2025. |
| Search concepts | The search combined three main concepts: total knee arthroplasty, remote or digital rehabilitation, and randomized controlled trials. |
| Controlled vocabulary | MeSH terms, Emtree terms, CINAHL subject headings, and CENTRAL MeSH terms were used where applicable. |
| Free-text terms | Free-text terms were used for TKA, remote/tele/digital rehabilitation, and randomized trials. |
| Field modifiers and truncation | Database-specific field modifiers and truncation were used where applicable. |
| CENTRAL trial filter | CENTRAL was not restricted by an additional randomized trial filter because CENTRAL primarily indexes controlled trials. |
| Date limits | No date restriction was applied. |
| Language limits | Studies published in English or Chinese were eligible because these were the languages that could be reliably assessed by the review team. |
| Supplementary searching | Reference lists of included studies and relevant systematic reviews were manually screened. |
| Grey literature | Grey literature databases were not searched because this review focused on peer-reviewed RCTs with extractable outcome data. |
| Trial registries | Trial registries were not searched because this was not part of the search methodology. |
| Conference proceedings | Conference proceedings were not searched because this was not part of the search methodology. |
| Citation searching | Citation searching was not performed as a separate search method. |
| Automated search tools | No automated search tools or text-mining tools were used. |
| Record management | All retrieved records were imported into EndNote 21 and Rayyan. |
| Deduplication | Duplicate records were removed before title and abstract screening. |
| Complete search strategies | The complete database-specific search strategies, including platform, search date, date range, limits, records retrieved, and exact search strings, are provided below. |

**2. Complete Database-Specific Search Strategies**

**2.1 PubMed (MEDLINE)**

Platform: PubMed

Search date: May 5, 2026

Date range: From inception to May 5, 2026

Limits: No date restriction; English or Chinese language eligibility was applied during screening

Records retrieved: 876

Exact search strategy:

#1

("Arthroplasty, Replacement, Knee"[Mesh]

OR "Knee Prosthesis"[Mesh]

OR arthroplast*[Title/Abstract]

OR "joint replacement*"[Title/Abstract]

OR "joint arthroplast*"[Title/Abstract]

OR "total knee arthroplasty"[Title/Abstract]

OR "total knee replacement"[Title/Abstract]

OR "knee arthroplasty"[Title/Abstract]

OR "knee replacement"[Title/Abstract]

OR "knee prosthesis"[Title/Abstract]

OR TKA[Title/Abstract]

OR TKR[Title/Abstract])

#2

("Telerehabilitation"[Mesh]

OR "Telemedicine"[Mesh]

OR "Remote Consultation"[Mesh]

OR "Mobile Applications"[Mesh]

OR "Videoconferencing"[Mesh]

OR "Therapy, Computer-Assisted"[Mesh]

OR telerehabilitation[Title/Abstract]

OR "tele-rehabilitation"[Title/Abstract]

OR telerehab*[Title/Abstract]

OR telehealth[Title/Abstract]

OR "tele-health"[Title/Abstract]

OR telemedicine[Title/Abstract]

OR "tele-medicine"[Title/Abstract]

OR telecare[Title/Abstract]

OR "tele-care"[Title/Abstract]

OR teleconsult*[Title/Abstract]

OR videoconferenc*[Title/Abstract]

OR "video consultation"[Title/Abstract]

OR "video conferencing"[Title/Abstract]

OR ehealth[Title/Abstract]

OR "e-health"[Title/Abstract]

OR mhealth[Title/Abstract]

OR "mobile health"[Title/Abstract]

OR "digital health"[Title/Abstract]

OR "digital rehabilitation"[Title/Abstract]

OR "digital physiotherapy"[Title/Abstract]

OR "digital intervention"[Title/Abstract]

OR "digital therapeutics"[Title/Abstract]

OR "internet-based"[Title/Abstract]

OR "internet intervention"[Title/Abstract]

OR "web-based"[Title/Abstract]

OR "web intervention"[Title/Abstract]

OR "online rehabilitation"[Title/Abstract]

OR "online physiotherapy"[Title/Abstract]

OR "online exercise"[Title/Abstract]

OR "online intervention"[Title/Abstract]

OR "online program"[Title/Abstract]

OR "remote rehabilitation"[Title/Abstract]

OR "remote physiotherapy"[Title/Abstract]

OR "remote monitoring"[Title/Abstract]

OR "remote care"[Title/Abstract]

OR "virtual rehabilitation"[Title/Abstract]

OR "virtual physiotherapy"[Title/Abstract]

OR "virtual care"[Title/Abstract]

OR "virtual therapy"[Title/Abstract]

OR "technology-assisted"[Title/Abstract]

OR "technology-based"[Title/Abstract]

OR "computer-assisted"[Title/Abstract]

OR "home-based monitoring"[Title/Abstract]

OR "self-management platform"[Title/Abstract]

OR smartphone*[Title/Abstract]

OR "mobile application*"[Title/Abstract]

OR app*[Title/Abstract]

OR apps[Title/Abstract]

OR wearable*[Title/Abstract]

OR software[Title/Abstract])

#3

(randomized controlled trial[Publication Type]

OR controlled clinical trial[Publication Type]

OR randomized[Title/Abstract]

OR randomised[Title/Abstract]

OR randomly[Title/Abstract]

OR trial[Title]

OR RCT[Title/Abstract])

#4

#1 AND #2 AND #3

**2.2 Cochrane Central Register of Controlled Trials (CENTRAL)**

Platform: Cochrane Library
Search date: May 5, 2026
Date range: From inception to May 5, 2026
Limits: No date restriction; English or Chinese language eligibility was applied during screening; no additional randomized trial filter was applied because CENTRAL primarily indexes controlled trials
Records retrieved: 1010
Exact search strategy:

#1

([mh "Arthroplasty, Replacement, Knee"]

OR [mh "Knee Prosthesis"]

OR (joint NEXT replacement*):ti,ab,kw

OR "total knee arthroplasty":ti,ab,kw

OR "total knee replacement":ti,ab,kw

OR "knee arthroplasty":ti,ab,kw

OR "knee replacement":ti,ab,kw

OR "knee prosthesis":ti,ab,kw

OR TKA:ti,ab,kw

OR TKR:ti,ab,kw)

#2

([mh Telerehabilitation]

OR [mh Telemedicine]

OR [mh "Remote Consultation"]

OR [mh "Mobile Applications"]

OR [mh Videoconferencing]

OR [mh "Therapy, Computer-Assisted"]

OR telerehabilitation:ti,ab,kw

OR "tele-rehabilitation":ti,ab,kw

OR telerehab*:ti,ab,kw

OR telehealth:ti,ab,kw

OR "tele-health":ti,ab,kw

OR telemedicine:ti,ab,kw

OR "tele-medicine":ti,ab,kw

OR telecare:ti,ab,kw

OR "tele-care":ti,ab,kw

OR teleconsult*:ti,ab,kw

OR videoconferenc*:ti,ab,kw

OR "video consultation":ti,ab,kw

OR "video conferencing":ti,ab,kw

OR ehealth:ti,ab,kw

OR "e-health":ti,ab,kw

OR mhealth:ti,ab,kw

OR "mobile health":ti,ab,kw

OR "digital health":ti,ab,kw

OR "digital rehabilitation":ti,ab,kw

OR "digital physiotherapy":ti,ab,kw

OR "digital intervention":ti,ab,kw

OR "digital therapeutics":ti,ab,kw

OR "internet-based":ti,ab,kw

OR "internet intervention":ti,ab,kw

OR "web-based":ti,ab,kw

OR "web intervention":ti,ab,kw

OR "online rehabilitation":ti,ab,kw

OR "online physiotherapy":ti,ab,kw

OR "online exercise":ti,ab,kw

OR "online intervention":ti,ab,kw

OR "online program":ti,ab,kw

OR "remote rehabilitation":ti,ab,kw

OR "remote physiotherapy":ti,ab,kw

OR "remote monitoring":ti,ab,kw

OR "remote care":ti,ab,kw

OR "virtual rehabilitation":ti,ab,kw

OR "virtual physiotherapy":ti,ab,kw

OR "virtual care":ti,ab,kw

OR "virtual therapy":ti,ab,kw

OR "technology-assisted":ti,ab,kw

OR "technology-based":ti,ab,kw

OR "computer-assisted":ti,ab,kw

OR "home-based monitoring":ti,ab,kw

OR "self-management platform":ti,ab,kw

OR smartphone*:ti,ab,kw

OR "mobile application*":ti,ab,kw

OR app:ti,ab,kw

OR apps:ti,ab,kw

OR wearable*:ti,ab,kw

OR software:ti,ab,kw)

#3

#1 AND #2

**2.3 Embase**

Platform: Ovid
Search date: May 5, 2026
Date range: From inception to May 5, 2026
Limits: No date restriction; English or Chinese language eligibility was applied during screening; randomized controlled trials were identified using Emtree terms and trial-related free-text terms
Records retrieved: 1730
Exact search strategy:

#1

exp knee arthroplasty/

OR exp knee prosthesis/

OR arthroplast*.ti,ab,kw.

OR joint replacement*.ti,ab,kw.

OR joint arthroplast*.ti,ab,kw.

OR total knee arthroplasty.ti,ab,kw.

OR total knee replacement.ti,ab,kw.

OR knee arthroplasty.ti,ab,kw.

OR knee replacement.ti,ab,kw.

OR knee prosthesis.ti,ab,kw.

OR TKA.ti,ab,kw.

OR TKR.ti,ab,kw.

#2

exp telerehabilitation/

OR exp telemedicine/

OR exp remote consultation/

OR exp mobile application/

OR exp digital health/

OR exp videoconferencing/

OR exp computer assisted therapy/

OR exp smartphone/

OR telerehabilitation.ti,ab,kw.

OR tele-rehabilitation.ti,ab,kw.

OR telerehab*.ti,ab,kw.

OR telehealth.ti,ab,kw.

OR tele-health.ti,ab,kw.

OR telemedicine.ti,ab,kw.

OR tele-medicine.ti,ab,kw.

OR telecare.ti,ab,kw.

OR tele-care.ti,ab,kw.

OR teleconsult*.ti,ab,kw.

OR videoconferenc*.ti,ab,kw.

OR video consultation.ti,ab,kw.

OR video conferencing.ti,ab,kw.

OR ehealth.ti,ab,kw.

OR e-health.ti,ab,kw.

OR mhealth.ti,ab,kw.

OR mobile health.ti,ab,kw.

OR digital health.ti,ab,kw.

OR digital rehabilitation.ti,ab,kw.

OR digital physiotherapy.ti,ab,kw.

OR digital intervention.ti,ab,kw.

OR digital therapeutics.ti,ab,kw.

OR internet-based.ti,ab,kw.

OR internet intervention.ti,ab,kw.

OR web-based.ti,ab,kw.

OR web intervention.ti,ab,kw.

OR online rehabilitation.ti,ab,kw.

OR online physiotherapy.ti,ab,kw.

OR online exercise.ti,ab,kw.

OR online intervention.ti,ab,kw.

OR online program.ti,ab,kw.

OR remote rehabilitation.ti,ab,kw.

OR remote physiotherapy.ti,ab,kw.

OR remote monitoring.ti,ab,kw.

OR remote care.ti,ab,kw.

OR virtual rehabilitation.ti,ab,kw.

OR virtual physiotherapy.ti,ab,kw.

OR virtual care.ti,ab,kw.

OR virtual therapy.ti,ab,kw.

OR technology-assisted.ti,ab,kw.

OR technology-based.ti,ab,kw.

OR computer-assisted.ti,ab,kw.

OR home-based monitoring.ti,ab,kw.

OR self-management platform.ti,ab,kw.

OR smartphone*.ti,ab,kw.

OR mobile application*.ti,ab,kw.

OR app*.ti,ab,kw.

OR wearable*.ti,ab,kw.

OR software.ti,ab,kw.

#3

exp randomized controlled trial/

OR exp controlled clinical trial/

OR exp randomization/

OR random*.ti,ab,kw.

OR randomly.ti,ab,kw.

OR trial.ti,ab,kw.

OR RCT.ti,ab,kw.

#4

#1 AND #2 AND #3

**2.4 Web of Science Core Collection**

Platform: Web of Science
Search date: May 5, 2026
Date range: From inception to May 5, 2026

Limits: No date restriction; English or Chinese language eligibility was applied during screening; randomized controlled trials were identified using trial-related free-text terms
Records retrieved: 395
Exact search strategy:

#1

TS=("total knee arthroplasty"

OR "total knee replacement"

OR "knee arthroplasty"

OR "knee replacement"

OR "knee prosthesis"

OR TKA

OR TKR)

#2

TS=(telerehabilitation

OR "tele-rehabilitation"

OR telerehab*

OR telehealth

OR "tele-health"

OR telemedicine

OR "tele-medicine"

OR telecare

OR "tele-care"

OR teleconsult*

OR videoconferenc*

OR "video consultation"

OR "video conferencing"

OR ehealth

OR "e-health"

OR mhealth

OR "mobile health"

OR "digital health"

OR "digital rehabilitation"

OR "digital physiotherapy"

OR "digital intervention"

OR "digital therapeutics"

OR "internet-based"

OR "internet intervention"

OR "web-based"

OR "web intervention"

OR "online rehabilitation"

OR "online physiotherapy"

OR "online exercise"

OR "online intervention"

OR "online program"

OR "remote rehabilitation"

OR "remote physiotherapy"

OR "remote monitoring"

OR "remote care"

OR "virtual rehabilitation"

OR "virtual physiotherapy"

OR "virtual care"

OR "virtual therapy"

OR "technology-assisted"

OR "technology-based"

OR "computer-assisted"

OR "home-based monitoring"

OR "self-management platform"

OR wearable*

OR smartphone*

OR software

OR app*)

#3

TS=(random*

OR randomly

OR trial

OR RCT)

#4

#1 AND #2 AND #3

**2.5 CINAHL (via EBSCOhost)**

Platform: EBSCOhost
Search date: May 5, 2026
Date range: From inception to May 5, 2026

Limits: No date restriction; English or Chinese language eligibility was applied during screening; randomized controlled trials were identified using CINAHL subject headings and trial-related free-text terms
Records retrieved: 126
Exact search strategy:

#1
(MH "Arthroplasty, Replacement, Knee+")
OR (MH "Knee Prosthesis+")
OR TI ("total knee arthroplasty"
OR "total knee replacement"
OR "knee arthroplasty"
OR "knee replacement"
OR "knee prosthesis"
OR TKA
OR TKR
)
OR AB (
"total knee arthroplasty"
OR "total knee replacement"
OR "knee arthroplasty"
OR "knee replacement"
OR "knee prosthesis"
OR TKA
OR TKR
)

#2
(MH "Telerehabilitation+")
OR (MH "Telemedicine+")
OR (MH "Remote Consultation+")
OR (MH "Mobile Applications+")
OR (MH "Videoconferencing+")
OR TI (
telerehabilitation
OR "tele-rehabilitation"
OR telerehab*
OR telehealth
OR "tele-health"
OR telemedicine
OR "tele-medicine"
OR telecare
OR "tele-care"
OR teleconsult*
OR videoconferenc*
OR "video consultation"
OR "video conferencing"
OR ehealth
OR "e-health"
OR mhealth
OR "mobile health"
OR "digital rehabilitation"
OR "digital physiotherapy"
OR "internet-based"
OR "web-based"
OR "online rehabilitation"
OR "online physiotherapy"
OR "remote rehabilitation"
OR "remote physiotherapy"
OR "remote monitoring"
OR "virtual rehabilitation"
OR "virtual physiotherapy"
OR wearable*
OR smartphone*
OR "mobile application*"
)
OR AB (
telerehabilitation
OR "tele-rehabilitation"
OR telerehab*
OR telehealth
OR "tele-health"
OR telemedicine
OR "tele-medicine"
OR telecare
OR "tele-care"
OR teleconsult*
OR videoconferenc*
OR "video consultation"
OR "video conferencing"
OR ehealth
OR "e-health"
OR mhealth
OR "mobile health"
OR "digital rehabilitation"
OR "digital physiotherapy"
OR "internet-based"
OR "web-based"
OR "online rehabilitation"
OR "online physiotherapy"
OR "remote rehabilitation"
OR "remote physiotherapy"
OR "remote monitoring"
OR "virtual rehabilitation"
OR "virtual physiotherapy"
OR wearable*
OR smartphone*
OR "mobile application*"
)

#3
(MH "Randomized Controlled Trials+")
OR TI (
random*
OR randomly
OR trial
OR RCT
OR "controlled clinical trial"
OR "randomized controlled trial"
OR "randomised controlled trial"
)
OR AB (
random*
OR randomly
OR trial
OR RCT
OR "controlled clinical trial"
OR "randomized controlled trial"
OR "randomised controlled trial"
)

#4
#1 AND #2 AND #3

**2.6 Scopus**

Platform: Scopus
Search date: May 5, 2026
Date range: From inception to May 5, 2026

Limits: No date restriction; English or Chinese language eligibility was applied during screening; randomized controlled trials were identified using trial-related free-text terms
Records retrieved: 931
Exact search strategy:

#1
TITLE-ABS-KEY(
"total knee arthroplasty"
OR "total knee replacement"
OR "knee arthroplasty"
OR "knee replacement"
OR "knee prosthesis"
OR TKA
OR TKR
)

#2
TITLE-ABS-KEY(
telerehabilitation
OR "tele-rehabilitation"
OR telerehab*
OR telehealth
OR telemedicine
OR telecare
OR teleconsult*
OR videoconferenc*
OR ehealth
OR mhealth
OR "mobile health"
OR "digital health"
OR "digital rehabilitation"
OR "digital physiotherapy"
OR "digital intervention"
OR "internet-based"
OR "internet intervention"
OR "web-based"
OR "web intervention"
OR "online rehabilitation"
OR "online physiotherapy"
OR "online intervention"
OR "remote rehabilitation"
OR "remote physiotherapy"
OR "remote monitoring"
OR "virtual rehabilitation"
OR "virtual physiotherapy"
OR "virtual care"
OR "virtual therapy"
OR wearable*
OR smartphone*
OR "mobile application*"
OR app-based
OR "app-based rehabilitation"
OR software
)

#3
TITLE-ABS-KEY(
random*
OR randomly
OR trial
OR RCT
OR "controlled clinical trial"
OR "randomized controlled trial"
OR "randomised controlled trial"
)

#4
#1 AND #2 AND #3

**2.7 CNKI (China National Knowledge Infrastructure)**

Platform: CNKI
Search date: May 5, 2026
Date range: From inception to May 5, 2026

Limits: No date restriction; English or Chinese language eligibility was applied during screening; randomized controlled trials were identified using trial-related Chinese search terms
Records retrieved: 86
Exact search strategy:

#1

SU = (“全膝关节置换”

OR “膝关节置换”

OR “人工膝关节置换”

OR “全膝置换”

OR “膝关节成形术”

OR “膝关节表面置换”

OR “关节置换”

OR “关节成形术”

OR “TKA”

OR “TKR”)

#2

SU = (“远程康复”

OR “远程医疗”

OR “远程康复治疗”

OR “远程物理治疗”

OR “远程护理”

OR “远程监测”

OR “互联网康复”

OR “网络康复”

OR “线上康复”

OR “在线康复”

OR “数字康复”

OR “数字医疗”

OR “数字干预”

OR “数字治疗”

OR “移动医疗”

OR “智慧医疗”

OR “虚拟康复”

OR “虚拟治疗”

OR “视频康复”

OR “视频随访”

OR “视频咨询”

OR “居家康复”

OR “居家监测”

OR “APP康复”

OR “手机应用”

OR “移动应用”

OR “网络干预”

OR “互联网干预”

OR “远程干预”

OR “技术辅助”

OR “可穿戴设备”

OR “智能设备”

OR “自我管理平台”)

#3

SU = (“随机对照试验”

OR “随机”

OR “随机分组”

OR “对照研究”

OR “临床试验”)

#4

#1 AND #2 AND #3
